# Hungarian population norms for the 15D generic preference-accompanied health status measure

**DOI:** 10.1007/s11136-023-03514-x

**Published:** 2023-09-14

**Authors:** Anna Nikl, Mathieu F. Janssen, Valentin Brodszky, Fanni Rencz

**Affiliations:** 1 Department of Health Policy, Corvinus University of Budapest, 8 Fővám tér, 1093 Budapest, Hungary; 2 Semmelweis University Doctoral School, Budapest, Hungary; 3 Section Medical Psychology and Psychotherapy, Department of Psychiatry, Erasmus MC, Rotterdam, The Netherlands

**Keywords:** 15D, Generic preference-accompanied measures, Self-reported health, Utility, Population norms, Hungary

## Abstract

**Objectives:**

15D is a generic preference-accompanied health status measure covering a wide range of health areas, including sensory functions. The aim of this study was to establish population norms for the 15D instrument in Hungary.

**Methods:**

2000 members of the Hungarian adult general population participated in an online cross-sectional survey in August 2021. The sample was broadly representative in terms of gender, age groups, highest level of education, geographical region, and settlement type. Index values were derived using the Norwegian 15D value set. In addition to providing population norms, mean index values were computed for 32 physical and 24 mental health condition groups.

**Results:**

Most respondents (78.7%) reported problems in at least one 15D domain. The most problems were reported with sleeping (50.7%), followed by vitality (49.2%), distress (43.6%), discomfort and symptoms (31.2%), depression (31.1%), sexual activities (29.6%), breathing (28.1%), and vision (27.8%). The mean 15D index value was 0.810. With advancing age categories, the 15D index values showed an inverse U-shaped curve. Generally, mean index values in respondents with mental health conditions were lower [range 0.299 (post-traumatic stress disorder) to 0.757 (smoking addiction)] than those of respondents with physical conditions [range 0.557 (liver cirrhosis) to 0.764 (allergies)].

**Conclusions:**

This study provided 15D population norms of the Hungarian general population; furthermore, this is the first study to provide population norms for the 15D in any country. The values established in this study can serve as benchmarks for evaluating efficacy outcomes in clinical trials, quantifying disease burden and identifying unmet needs.

**Supplementary Information:**

The online version contains supplementary material available at 10.1007/s11136-023-03514-x.

## Introduction

In health technology assessment of new health interventions and policy planning, decision-makers take into account the health of different patient groups as well as the general population. Health status can be measured with various instruments, which are divided into two categories: generic and disease-specific measures [1, 2]. Disease-specific instruments consist of health domains relevant for patients with a specific disease, while generic instruments have domains relevant across multiple patient populations as well as the general population (e.g., physical and mental health) [3, 4]. Preference-accompanied measures (PAMs) are a type of generic health status measure that usually comprise a descriptive system and a value set of preference weights for all the possible health profiles defined by the descriptive system [5]. The value set allows the estimation of index values that can be used to quantify quality-adjusted life-years used in cost-utility analyses [6].

Although the EQ-5D is the most widely used generic PAM at an international level, it might not be able to capture all relevant areas of health due to its brevity [7–9]. A more lengthy generic PAM is the 15D, developed in the early 1970s in Finland, which assesses health with a 15-dimensional descriptive system [10]. According to PubMed, more than 500 publications are available with the 15D from the past 25 years. The questionnaire has been translated into 32 languages; however, it is mainly used in Nordic countries [11]. Currently, country-specific value sets are available for three countries: Finland [12], Denmark [13], and two for Norway estimated with different methods [14, 15]. The validity, reliability, and responsiveness of the 15D have been established in numerous health conditions and populations, such as chronic obstructive pulmonary disease [16], epilepsy [17], HIV/AIDS [18], musculoskeletal, cardiovascular and psychosomatic disorders [19], critical care [20], visual impairment [21], elderly [22], Parkinson’s disease [23], cardiac surgery [24, 25], chronic pain [26], and pelvic organ prolapse surgery [27].

The responses collected by various generic and disease-specific health status measures are frequently interpreted using population norms, otherwise known as population reference data. These norms facilitate the assessment of disease burden by comparing patients’ health to the age- and gender-matched general population [28]. They may also serve the purpose of monitoring the change in the general population’s health and as benchmarks for evaluating efficacy outcomes in clinical trials and identifying unmet needs. In Hungary, population reference data have been established for the EQ-5D-3L [29], 36-Item Short Form Survey (SF-36) [30], and for two adult profile measures of the Patient-Reported Outcomes Measurement Information System (PROMIS-29 + 2 and PROMIS Global Health) [31, 32] generic health status measures, as well as for the Dermatology Life Quality Index (DLQI) disease-specific measure [33].

The Hungarian version of the 15D showed broadly good psychometric properties, including convergent validity with the EQ-5D-5L and known-group validity across multiple health condition groups [34]. However, 15D population reference data have not been established in Hungary (or in any other countries) yet. Given that the 15D is a comprehensive PAM, covering a wide range of health including sensory functions, it offers a strong basis to describe the general population’s health status. Therefore, the primary objective of this study is to establish Hungarian population norms for the 15D by gender and age. In addition, we assess its association with sociodemographic characteristics, such as gender, age, and other factors, as well as several chronic physical and mental health conditions. We also provide index value estimates for a wide array of prevalent chronic diseases, including physical and mental health conditions.

## Methods

### Study design

The cross-sectional data used in this study were collected within the framework of a larger survey aiming to assess the health status of the Hungarian population with a particular focus on mental health [34]. The project was approved by the Research Ethics Committee of the Corvinus University of Budapest (no. KRH/166/2021). In August 2021, a total of 2000 members of the Hungarian adult general population (18 or older) were recruited from an online panel. Members of the general population may register voluntarily to the panel to complete surveys in exchange for survey points that can be later redeemed for gift cards or prize lottery tickets. All respondents were asked to give informed consent before starting the questionnaire. The study sample aimed for a broad representativeness of the general population in terms of gender, age group, highest level of education, geographical region, and settlement type [35].

Participants completed a self-administered online survey comprising a selection of standardized questionnaires, including the validated Hungarian version of the 15D. Questions were related to the respondents’ sociodemographic characteristics, well-being, health status, and physical and mental health conditions. The latter were asked in two questions. First, respondents were presented with a list of chronic physical and mental conditions and asked if they experienced any self-reported physical and mental health conditions in the past 12 months. Then, they had to select those conditions that were diagnosed by a physician. Participants had the option to report the presence of both physical and mental health conditions. The physical health conditions on this list were based on the European Health Interview Survey (EHIS) in Hungary in 2019, which was extended by a few other prevalent chronic conditions [36]. The fifth edition of the Diagnostic and Statistical Manual of Mental Disorders (DSM-5) served as a basis for the mental health conditions on the list [37].

### The 15D instrument

The 15D comprises the following 15 domains of health: mobility, vision, hearing, breathing, sleeping, eating, speech, excretion, usual activities, mental function, discomfort and symptoms, depression, distress, vitality, and sexual activities [10]. Respondents are asked to recall their present health status on a five-point response scale for each domain. The response levels can be capability (e.g., I can hear normally, i.e. normal speech (with or without a hearing aid). / I hear normal speech with a little difficulty. / I hear normal speech with considerable difficulty; in conversation I need voices to be louder than normal. / I hear even loud voices poorly; I am almost deaf. / I am completely deaf.) or severity type scales (e.g., I have no/mild/marked/severe/unbearable physical discomfort or symptoms, e.g. pain, ache, nausea, itching etc.), varying per domain. Responses from the 15 domains can be combined into a 15-digit string expressing a health profile. Overall, 5^15^ (more than 30 billion) health profiles can theoretically be described by the instrument.

The 15D index value can be computed based on these health profiles by applying a formula that assigns values (weights) to each response level in each health domain. The index value can then be calculated by subtracting the corresponding values from 1, where 1 refers to full health. To calculate the 15D index values, we used the Norwegian value set [15]. The index values of the Norwegian value set range from -0.516 to 1, where negative values describe health states worse than dead. The Norwegian value set was selected as it is the most recently developed one that compared to previous 15D valuation studies, benefited more from the most recent valuation and modelling advancements.

### Statistical analysis

The relative frequency of responses on each response level of each domain was calculated for the total sample and stratified by gender and age groups. We dichotomized responses (‘no problems’ or ‘any problems’) in each domain, then used Pearson’s χ^2^ tests to detect any differences between the frequency of respondents across these subgroups.

Mean level scores (LS) were also calculated to summarize the responses on each 15D domain according to gender and age groups. To compute LS, we transformed 1–5 responses on each domain to a 0–100 scale, where higher scores indicate worse health status [38]. Mean and 95% confidence intervals were computed for the 15D index values. Both for LS and index values, differences between sociodemographic subgroups were examined by Student’s *t* test and analysis of variance, where applicable. Mean index values were calculated for 32 physical and 24 mental health condition groups.

Multivariate linear regressions were used to explore the association of sociodemographic and health-related variables with the 15D index values. Homoskedasticity was evaluated by the Breusch-Pagan test. In case heteroskedasticity was present in the model, a correction using robust standard errors was performed. Gender, age, highest level of education, settlement type, geographical region, employment status, marital status, household’s per capita net monthly income, and physical and mental health conditions with a sample size of at least 30 cases were included in the models as independent variables. All independent variables were categorical. The household’s per capita net monthly income was split according to the median income level (112,500 HUF).

All statistical analyses were carried out using R Statistical Software (version 4.1.1; R Foundation for Statistical Computing, Vienna, Austria). All statistics were two-sided, and the significance level was set at 0.05.

## Results

### Characteristics of the study population

A target sample size of 2000 respondents was achieved with a response rate of 77.8%. The main characteristics of the study sample are presented in Table 1. The mean age was 46.3 (SD = 16.9) and 57.3% were female. The composition of the sample reasonably approximated that of the Hungarian general population. However, persons with secondary education were slightly underrepresented and at the same time, those with tertiary were overrepresented. The 25–34 age group was somewhat overrepresented as well. Almost two-thirds of the study sample, 1261 participants reported chronic physical conditions (63.1%) and 703 reported mental health conditions (35.2%) diagnosed by a physician, resulting in 1429 respondents with chronic illness, which accounts for 71.5% of the sample.

**Table 1:** Mean 15D index values according to sociodemographic and health-related characteristics

Variables	Reference population (%)^a^	N	%	15D index value		
				Mean	95% CI	*p* value^b^
Total	100	2000	100	0.810	0.800–0.819	–
*Gender*						
Male	46.9	855	42.8	0.820	0.805–0.835	0.0711
Female	53.1	1145	57.3	0.802	0.789–0.815	
*Age groups (years)*						
18–24	10.0	202	10.1	0.782	0.741–0.822	0.1286
25–34	15.2	441	22.1	0.823	0.801–0.844	
35–44	19.5	337	16.9	0.819	0.795–0.843	
45–54	16.0	285	14.3	0.825	0.802–0.848	
55–64	16.8	337	16.9	0.803	0.781–0.826	
65 and above	22.5	398	19.9	0.796	0.777–0.815	
*Highest level of education*						
Primary	23.8	544	27.2	0.775	0.753–0.796	< 0.0001
Secondary	55.0	909	45.5	0.807	0.792–0.822	
Tertiary	21.2	547	27.4	0.849	0.835–0.863	
*Settlement type*						
Capital	17.9	390	19.5	0.825	0.806–0.845	0.0003
City	52.6	979	49.0	0.822	0.809–0.835	
Village	29.5	631	31.6	0.781	0.761–0.800	
*Geographical region*						
Central Hungary	30.4	619	31.0	0.811	0.794–0.827	0.9850
Great Plain and North	30.2	790	39.5	0.810	0.794–0.825	
Transdanubia	39.5	591	29.6	0.809	0.790–0.827	
*Employment status*						
Employed	53.1	1074	53.7	0.827	0.814–0.840	< 0.0001
Retired	26.1	502	25.1	0.805	0.789–0.822	
Disability pensioner	3.1	55	2.8	0.559	0.486–0.631	
Student	3.1	68	3.4	0.853	0.807–0.900	
Unemployed	4.7	91	4.6	0.792	0.748–0.836	
Homemaker/housewife	1.0	49	2.5	0.801	0.746–0.857	
Other	8.9	161	8.1	0.787	0.745–0.830	
*Marital status*						
Married	45.6	825	41.3	0.835	0.822–0.848	< 0.0001
Domestic partnership	13.4	417	20.9	0.834	0.814–0.853	
Single	18.5	472	23.6	0.767	0.743–0.791	
Widowed	11.4	129	6.5	0.780	0.741–0.819	
Divorced	11.1	157	7.9	0.766	0.729–0.803	
*Household’s per capita net monthly income (HUF)*^*c*^						
1st quintile (≤ 75,000.3)	N/A	300	15.0	0.751	0.720–0.781	< 0.0001
2nd quintile (75,000.3 & ≤ 112,500.5)	N/A	377	18.8	0.786	0.763–0.808	
3rd quintile (> 112,500.5 & ≤ 142,500.3)	N/A	295	14.8	0.808	0.785–0.831	
4th quintile (> 142,500.3 & ≤ 212,500.5)	N/A	373	18.6	0.828	0.808–0.848	
5th quintile (> 212,500.5)	N/A	275	13.8	0.834	0.810–0.858	
*Diagnosis of any chronic disease*^*c,d*^						
Mental	48.0	168	8.4	0.795	0.754–0.835	< 0.0001
Physical		726	36.3	0.842	0.830–0.853	
Both		535	26.8	0.698	0.678–0.717	
None	52.0	406	20.3	0.903	0.884–0.922	

CI confidence intervals

^a^Hungarian Central Statistical Office: Microcensus 2016

^b^Difference in index values between groups is tested by Student’s *t* test (two groups) or analysis of variance (three or more groups)

^c^The number of respondents who responded ‘do not know’ or refused to answer was n = 380 (19.0%) for the household’s per capita net monthly income and n = 165 (8.3%) for the diagnosis of any chronic disease

^d^Hungarian Central Statistical Office: European Health Interview Survey in Hungary, 2019

Totals may not add up to 100 by groups due to rounding. N/A = not available

### Health problems by 15D domains

**Fig. 1:**
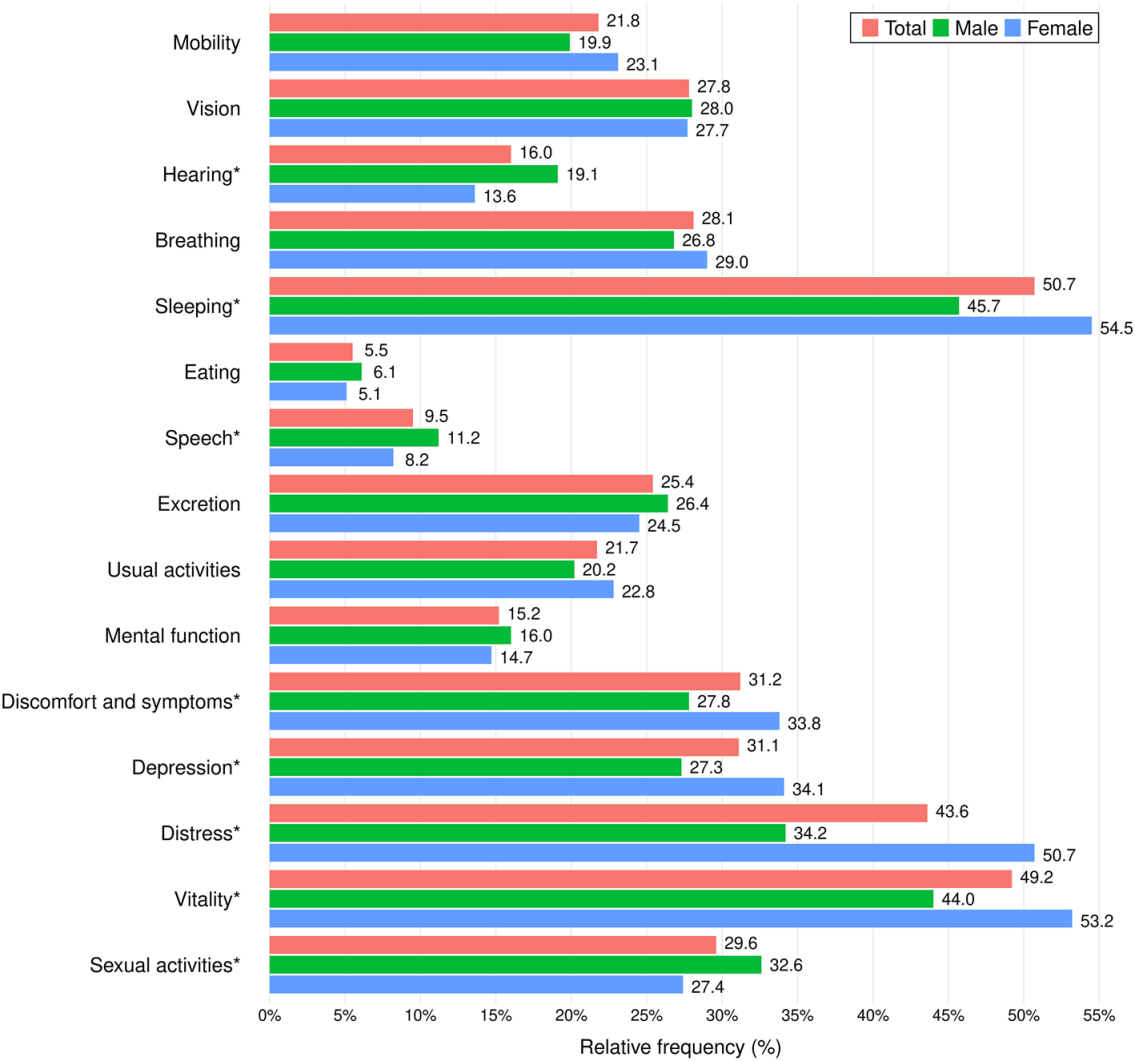
Proportion of respondents reporting any problems in 15D domains. Pearson’s χ^2^ test was performed to assess the difference in the proportion of problems between genders. All domains where *p* value was < 0.05 are marked with asterisks

The majority of the study population (78.7%) reported having problems in at least one 15D domain. Respondents experienced the least problems in eating (5.5%), then in speech (9.5%) and mental function (15.2%), while sleeping problems were the most frequently reported affecting 50.7% of the population, followed by vitality (49.2%) and distress (43.6%). Comparing the responses by gender, females had significantly more problems with distress than males (50.7% vs. 34.2%), as well as vitality (53.2% vs. 44.0%), sleeping (54.5% vs. 45.7%), depression (34.1% vs. 27.3%), and discomfort and symptoms (33.8% vs. 27.8%). On the other hand, females had significantly fewer issues with hearing (13.6% vs. 19.1%), sexual activities (27.4% vs. 32.6%), and speech (8.2% vs. 11.2%). The difference between the two genders was insignificant for mobility, vision, breathing, eating, excretion, usual activities, and mental function (Fig. 1).

**Fig. 2:**
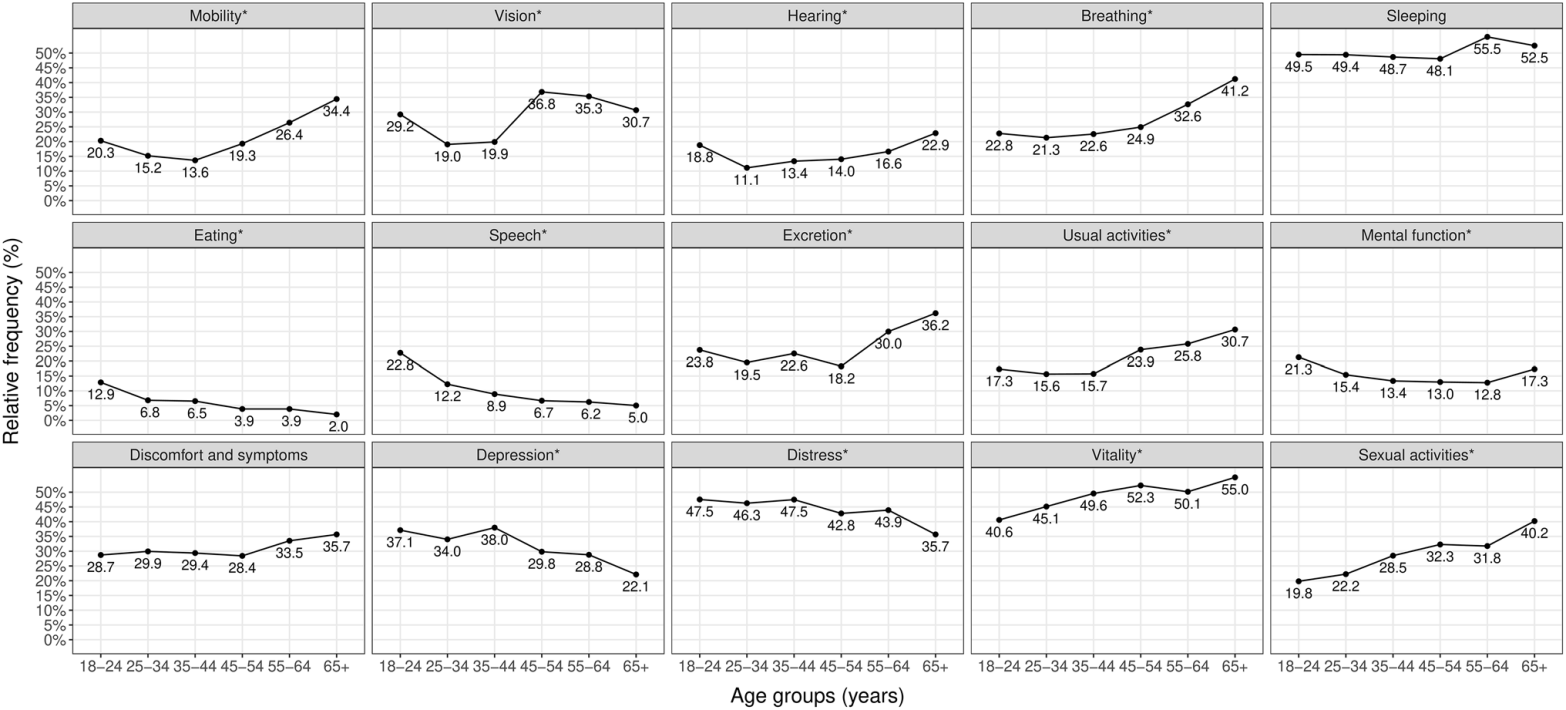
Proportion of respondents reporting any problems in each domain by age groups. Pearson’s χ^2^ test was performed to assess the difference between age groups. All domains where *p* value was < 0.05 are marked with asterisks

In general, the least problems in all age groups were found with eating, ranging from 2.0% (65-year-olds or more) to 12.9% (18–24-year-olds), while respondents reported the most problems with sleeping for the 18–24- (49.5%), 25–34- (49.4%) and 55–64-year-olds (55.5%), and vitality for the 35–44- (49.6%), 45–54- (52.3%), as well as the at least 65-year-olds (55.0%). Problems tended to increase with age in the mobility, vision, hearing, breathing, excretion, usual activities, vitality, and sexual activities domains. Problems decreased with age in the eating, speech, mental function, depression, and distress domains. The difference between the age groups was insignificant for the sleeping and discomfort and symptoms domains (Fig. 2).

**Fig. 3:**
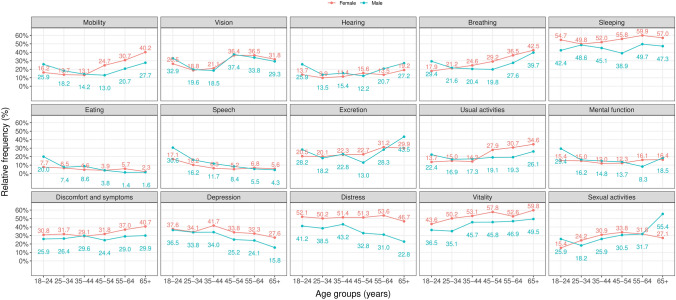
Proportion of respondents reporting any problems in each domain by age and gender groups

When comparing gender and age groups, both males and females in every age group had the least problems with eating (Fig. 3). As for males, the 18–24-, 25–34-, and 55–64-year-olds had the most problems with sleeping, the 35–44- and 45–54-year-olds with vitality, and the 65-year-olds or more with sexual activities. In comparison, the 18–24 and 55–64-year-old females experienced the most problems with sleeping, while the 25–34, 35–44-, 45–54-, and 65-year-olds or more with vitality, as well as the 25–34-year-olds also with distress. Online resources 1–3 present the responses on each 15D domain in different age groups for all participants, then separately for males and females.

Summary data of mean LS are presented in Online resources 4–6. In the total sample, respondents had the highest mean LS in vitality (18.1), while the lowest mean LS in eating (2.3). As for genders, females had significantly higher mean LS than males in distress (19.2 vs. 12.6), sleeping (19.8 vs. 15.7), vitality (19.7 vs. 16.0), discomfort and symptoms (11.8 vs. 9.7), depression (12.7 vs. 10.7), and breathing (10.5 vs. 8.8), while lower mean LS in sexual activities (11.8 vs. 15.3) and hearing (5.1 vs. 6.6). When comparing these results with the relative frequency of problems, differences between the two genders were found to be significant for both indicators in hearing, sleeping, discomfort and symptoms, depression, distress, vitality, and sexual activities health domains. Where females had more problems, they also had a higher mean LS. There was no significant difference between the relative frequency of problems between the two genders in breathing; however, males had a higher mean LS. Likewise, males had more problems with speech than females, but the difference in their mean LS was insignificant.

### Mean index values by sociodemographic and health-related characteristics

The mean 15D index value was 0.810 (95% CI 0.800–0.819), and 0.8% of the sample was in the negative range. Differences in index values between subgroups were insignificant for gender, age groups, and geographical region (Table 1). Respondents with a higher level of education had significantly higher mean 15D index values, as well as those living in the capital or larger cities, living in a domestic partnership or marriage, and those with higher net income per capita in their households. As for employment status, students had the highest average index values, followed by employed, then retired respondents, and homemakers/housewives, while disability pensioners had the lowest mean index value.

The mean 15D index values by age and gender are summarized in Table 2. Regarding women, no trend-like relationship can be discovered with advancing age; however, in the case of men, that relationship is somewhat inverse U-shaped.

**Table 2:** Mean 15D index values by gender and age groups

Age groups	Total				Males				Females			
	n	%	15D index values		n	%	15D index values		n	%	15D index values	
			Mean	95% CI			Mean	95% CI			Mean	95% CI
18–24	202	10.1	0.782	0.741–0.822	85	9.9	0.741	0.667–0.816	117	10.2	0.811	0.767–0.855
25–34	441	22.1	0.823	0.801–0.844	148	17.3	0.822	0.783–0.860	293	25.6	0.823	0.798–0.849
35–44	337	16.9	0.819	0.795–0.843	162	18.9	0.824	0.788–0.860	175	15.3	0.814	0.782–0.846
45–54	285	14.3	0.825	0.802–0.848	131	15.3	0.857	0.826–0.888	154	13.4	0.798	0.764–0.832
55–64	337	16.9	0.803	0.781–0.826	145	17.0	0.837	0.808–0.865	192	16.8	0.778	0.745–0.811
65 and above	398	19.1	0.796	0.777–0.815	184	21.5	0.812	0.786–0.837	214	18.7	0.783	0.755–0.810
Total	2000	100.0	0.810	0.800–0.819	855	100.0	0.820	0.805–0.835	1145	100.0	0.802	0.789–0.815

*CI* confidence intervals

Mean index values by different physical and mental health conditions are presented in Table 3. Healthy respondents had the highest mean index value (0.903). Among the physical conditions, respondents with allergies (0.764), hypertension (0.754), and thyroid diseases (0.744) had the highest 15D index values, while those with stroke (0.567), gastric or duodenal ulcer (0.561), and liver cirrhosis (0.557) had the lowest. In contrast to physical health conditions, participants with mental health conditions had significantly lower mean 15D index values (0.781 vs. 0.721, *p* < 0.0001). Among mental conditions, the higher mean values were reported in respondents smoking (0.757), having other addictions (0.717), and gambling addiction (0.684), while the lowest values were reported in attention deficit hyperactivity disorder (0.315), autism spectrum disorder (0.311) and post-traumatic stress disorder (0.299).

**Table 3:** Mean 15D index values according to chronic health conditions

Variables	N	%	Mean	95% CI
Healthy	406	20.3	0.903	0.884–0.922
***Physical health conditions***	***1261***	***63.1***	***0.781***	***0.769–0.792***
Allergies	332	16.6	0.764	0.741–0.788
Hypertension	551	27.6	0.754	0.735–0.772
Thyroid diseases	178	8.9	0.744	0.711–0.777
Atopic dermatitis	56	2.8	0.731	0.661–0.802
Psoriasis	53	2.7	0.728	0.665–0.791
Diabetes	218	10.9	0.727	0.694–0.759
Other physical health conditions	97	4.9	0.717	0.676–0.758
Other skin diseases	44	2.2	0.715	0.644–0.785
Gastroesophageal reflux disease	194	9.7	0.715	0.682–0.747
Musculoskeletal diseases	483	24.2	0.713	0.693–0.733
Hyperlipidaemia	252	12.6	0.712	0.682–0.741
Benign prostate hyperplasia	90	4.5	0.711	0.666–0.757
Cataract	85	4.3	0.707	0.661–0.753
Asthma	119	6.0	0.701	0.659–0.742
Chronic bronchitis, emphysema, COPD	101	5.1	0.701	0.656–0.747
Acne	37	1.9	0.696	0.615–0.777
Hearing impairment	136	6.8	0.682	0.639–0.725
Cancer, leukaemia, lymphoma	50	2.5	0.676	0.603–0.749
Heart attack	37	1.9	0.676	0.587–0.765
Headache, migraine	147	7.4	0.674	0.631–0.717
Glaucoma	32	1.6	0.670	0.590–0.751
Inflammatory bowel disease	38	1.9	0.665	0.590–0.739
Coronary artery disease, angina	58	2.9	0.651	0.586–0.715
Chronic kidney disease	30	1.5	0.647	0.555–0.739
Arrhythmias	178	8.9	0.642	0.607–0.678
Urinary incontinence	74	3.7	0.625	0.560–0.689
Visual impairment	171	8.6	0.618	0.580–0.655
Other heart disease	75	3.8	0.612	0.547–0.676
Epilepsy	17	0.9	0.578	0.424–0.732
Stroke	34	1.7	0.567	0.470–0.664
Gastric or duodenal ulcer	40	2.0	0.561	0.467–0.656
Liver cirrhosis	14	0.7	0.557	0.343–0.772
***Mental health conditions***	***703***	***35.2***	***0.721***	***0.703–0.739***
Smoking addiction	406	20.3	0.757	0.734–0.781
Other addictions	10	0.5	0.717	0.573–0.860
Gambling addiction	58	2.9	0.684	0.601–0.767
Alcohol addiction	79	4.0	0.646	0.579–0.712
Generalized anxiety disorder	307	15.4	0.645	0.614–0.676
Sleeping disorders	178	8.9	0.620	0.582–0.658
Learning disability	30	1.5	0.607	0.462–0.752
Substance addiction	24	1.2	0.587	0.422–0.752
Sexual disorder	40	2.0	0.567	0.477–0.657
Panic disorder	115	5.8	0.564	0.514–0.615
Eating disorder	27	1.4	0.560	0.424–0.696
Prescription drug addiction	56	2.8	0.545	0.452–0.638
Bipolar depression	35	1.8	0.529	0.426–0.633
Unipolar major depression	28	1.4	0.522	0.411–0.633
Phobia	49	2.5	0.492	0.393–0.590
Dysthymia	64	3.2	0.475	0.411–0.539
Impulse-control disorder	15	0.8	0.443	0.265–0.622
Personality disorder	31	1.6	0.421	0.309–0.532
Dementia	18	0.9	0.373	0.230–0.515
Psychotic disorders	17	0.9	0.371	0.171–0.572
Obsessive compulsive disorder	21	1.1	0.360	0.216–0.505
Attention deficit hyperactivity disorder	11	0.6	0.315	0.074–0.556
Autism spectrum disorder	11	0.6	0.311	0.044–0.579
Post-traumatic stress disorder	14	0.7	0.299	0.115–0.483

CI confidence intervals, COPD chronic obstructive pulmonary disease

Participants could report having both physical and mental health conditions

### Multivariate linear regression of 15D index values

Table 4 shows the results of the multivariate linear regression of 15D index values. Higher index values were associated with advancing age categories, reaching their highest in the 45–54 age group, then the value gradually decreased in the older age groups, revealing an inverse U-shaped curve. Respondents with a higher level of education had higher index values. Regarding employment status, disability pensioners’ index value was significantly lower than those of being employed, while students’ index value was higher. Respondents being married or in a domestic partnership also had higher index values as opposed to being single. Gender was not associated with the index value. Settlement type, geographical region, being retired, unemployed, homemaker/housewife, or other, being widowed or divorced, as well as household’s per capita net monthly income were also insignificant in the model. Eight of the 30 physical health conditions (hypertension, musculoskeletal diseases, hyperlipidaemia, diabetes, arrhythmias, visual impairment, hearing impairment, and asthma) were significantly associated with the 15D index values. Among these conditions, the largest index value decrement was associated with visual impairment (beta = − 0.067) and the smallest with hypertension (beta = − 0.021). Considering the mental health conditions, seven of the 13 (generalized anxiety disorder, panic disorder, alcohol addiction, prescription drug addiction, phobia, sexual disorder, and personality disorder) were associated with the 15D index value, where personality disorder had the largest (beta = − 0.121) and panic disorder the smallest (beta = − 0.057) impact. In line with previous results, mental health conditions were associated with a larger decrement in the index value, on average, than physical health conditions.

**Table 4:** Multivariate linear regression of 15D index values

Variables	Coefficient	95% CI	*p* value
Intercept	0.799	0.744, 0.854	< 0.0001
*Gender*			
Male^a^	–	–	–
Female	− 0.005	− 0.025, 0.014	0.5834
*Age groups (years)*			
18–24	–	–	–
25–34	0.050	0.000, 0.100	0.0498
35–44	0.077	0.025, 0.129	0.0035
45–54	0.090	0.039, 0.142	0.0006
55–64	0.089	0.035, 0.144	0.0014
65 and above	0.075	0.014, 0.136	0.0165
*Highest level of education*			
Primary	− 0.028	− 0.052, − 0.003	0.0253
Secondary	− 0.018	− 0.035, 0.000	0.0512
Tertiary^a^	–	–	–
*Settlement type*			
Capital^a^	–	–	–
City	− 0.004	− 0.035, 0.028	0.8213
Village	− 0.024	− 0.058, 0.009	0.1575
*Geographical region*			
Central Hungary^a^	–	–	–
Great Plain and North	0.022	− 0.006, 0.050	0.1264
Transdanubia	0.020	− 0.011, 0.050	0.2042
*Employment status*			
Employed^a^	–	–	–
Retired	0.019	− 0.010, 0.048	0.1890
Disability pensioner	− 0.109	− 0.161, − 0.057	< 0.0001
Student	0.076	0.013, 0.138	0.0171
Unemployed	− 0.006	− 0.046, 0.034	0.7567
Homemaker/housewife	0.020	− 0.021, 0.062	0.3381
Other	− 0.009	− 0.050, 0.032	0.6748
*Marital status*			
Single^a^	–	–	–
Married	0.050	0.023, 0.077	0.0003
Domestic partnership	0.064	0.036, 0.092	< 0.0001
Widowed	0.017	− 0.032, 0.066	0.4961
Divorced	0.037	− 0.003, 0.077	0.0697
*Household’s per capita net monthly income (HUF)*			
Lower median (≤ 112,500)^a^	–	–	–
Upper median (> 112,500)	0.003	− 0.019, 0.025	0.7944
Refused to answer	0.017	− 0.009, 0.043	0.2072
*Physical health conditions*^*b*^			
Hypertension	− 0.021	− 0.040, − 0.003	0.0223
Musculoskeletal diseases	− 0.051	− 0.069, − 0.033	< 0.0001
Allergies	0.005	− 0.015, 0.026	0.6226
Hyperlipidaemia	− 0.031	− 0.054, − 0.009	0.0061
Diabetes	− 0.027	− 0.053, − 0.001	0.0413
Gastroesophageal reflux disease	0.003	− 0.023, 0.030	0.8044
Thyroid diseases	0.013	− 0.011, 0.038	0.2863
Arrhythmias	− 0.053	− 0.083, − 0.023	0.0006
Visual impairment	− 0.067	− 0.100, − 0.034	0.0001
Headache, migraine	− 0.030	− 0.063, 0.002	0.0671
Hearing impairment	− 0.041	− 0.071, − 0.010	0.0092
Asthma	− 0.056	− 0.092, − 0.021	0.0020
Chronic bronchitis, emphysema, COPD	0.000	− 0.039, 0.039	0.9876
Other physical health conditions	− 0.036	− 0.079, 0.007	0.1014
Benign prostate hyperplasia	− 0.020	− 0.057, 0.017	0.2935
Cataract	− 0.015	− 0.057, 0.027	0.4788
Other heart disease	− 0.042	− 0.092, 0.008	0.0997
Urinary incontinence	− 0.045	− 0.091, 0.001	0.0578
Coronary artery disease, angina	− 0.029	− 0.090, 0.032	0.3490
Atopic dermatitis	0.023	− 0.024, 0.070	0.3348
Psoriasis	− 0.021	− 0.068, 0.026	0.3769
Cancer, leukaemia, lymphoma	− 0.021	− 0.076, 0.034	0.4567
Other skin diseases	0.008	− 0.054, 0.069	0.8012
Gastric or duodenal ulcer	− 0.041	− 0.107, 0.025	0.2201
Inflammatory bowel disease	0.008	− 0.051, 0.068	0.7807
Acne	0.011	− 0.055, 0.077	0.7369
Heart attack	0.019	− 0.057, 0.094	0.6245
Stroke	− 0.054	− 0.139, 0.030	0.2050
Glaucoma	− 0.013	− 0.096, 0.069	0.7514
Chronic kidney disease	0.053	− 0.019, 0.124	0.1474
*Mental health conditions*^*b*^			
Smoking addiction	− 0.011	− 0.030, 0.009	0.2735
Generalized anxiety disorder	− 0.107	− 0.137, − 0.078	< 0.0001
Sleeping disorders	− 0.036	− 0.072, 0.000	0.0524
Panic disorder	− 0.057	− 0.102, − 0.012	0.0125
Alcohol addiction	− 0.058	− 0.111, − 0.005	0.0309
Dysthymia	− 0.050	− 0.114, 0.015	0.1316
Gambling addiction	− 0.050	− 0.116, 0.016	0.1373
Prescription drug addiction	− 0.108	− 0.185, − 0.031	0.0059
Phobia	− 0.095	− 0.177, − 0.012	0.0240
Sexual disorder	− 0.086	− 0.157, − 0.015	0.0175
Bipolar depression	− 0.006	− 0.094, 0.081	0.8886
Personality disorder	− 0.121	− 0.231, − 0.012	0.0296
Learning disability	− 0.005	− 0.104, 0.093	0.9198

*CI* confidence intervals, *COPD* chronic obstructive pulmonary disease

^a^Reference category. The normative category, or the category which is at one of the ends was chosen as reference category

^b^No reported condition was considered as reference category

## Discussion

This study provided population norms for the 15D, estimated on a broadly representative sample of the adult Hungarian population. This is the first study to establish population reference values for the 15D instrument in any country and report 15D index values for over 55 chronic diseases, including physical and mental conditions. More than three-quarters of the respondents indicated having at least some health problems on the 15D, the most commonly reported ones being sleeping, vitality, and distress. A multivariate linear regression model was also estimated, controlling for different sociodemographic factors and several chronic health condition groups. A higher level of education was associated with a higher average 15D index value. Disability pensioners had lower and students had higher index values than employed participants and those being married or in a domestic partnership than single respondents. Altogether, 8/30 physical health conditions and 7/13 mental health conditions were significantly associated with the 15D index value.

It is worthwhile to compare our findings to previously established population norms for other generic health status measures in Hungary. The Hungarian SF-36 [30] and EQ-5D-3L [29] population norm studies were conducted more than two decades ago. Over the past 25 years, the population’s health status has likely changed due to various political, social, economic, technological and public health events, which include significant advancements in healthcare, as well as challenges such as an economic crisis and the COVID-19 pandemic. It is therefore more meaningful to compare the current results with the recently published Hungarian population reference values of the PROMIS-29 + 2 [31, 39]. Six out of the eight PROMIS-29 + 2 health domains (physical function, anxiety, depression, fatigue, sleep disturbance, and cognitive function) and the 0–10 pain intensity numeric rating scale in PROMIS-29 + 2 broadly correspond to seven 15D domains (mobility, distress, depression, vitality, sleeping, discomfort and symptoms, and mental function). A substantially higher proportion of respondents reported any problems in all PROMIS-29 + 2 domains and also on the pain intensity scale. The largest difference can be observed in the PROMIS-29 + 2 cognitive function and 15D mental function (63.5% vs. 15.2%), while the smallest in the PROMIS-29 + 2 physical function and 15D mobility pair (39.3% vs. 21.8%). Considering health status by age group, the results of this study are somewhat consistent with the results of the study conducted for PROMIS-29 + 2. Neither the PROMIS-29 + 2 sleep disturbance nor the 15D sleeping domain revealed any differences between the age groups. Problems tended to decrease with age in PROMIS-29 + 2 depression, anxiety, fatigue, and cognitive function, as well as 15D depression, distress, and mental function domains, while older generations had more problems in 15D vitality. Problems rose with age for PROMIS-29 + 2 physical function and pain intensity, along with 15D mobility, whereas no difference was found between age groups in 15D discomfort and symptoms. The 15D most likely underestimates pain as it differs from most other PAMs, such as the PROMIS-29 + 2, in that the word ‘pain’ is not included in the domain heading (i.e., discomfort and symptoms), but appears only among the examples provided in the response levels (I have no/mild/marked/severe/unbearable physical discomfort or symptoms, e.g. pain, ache, nausea, itching etc.). Another difference between the two instruments is the recall period; for the 15D, respondents are asked to report their present health status, whereas, for the PROMIS-29 + 2, the recall period is either unspecified or refers to the past seven days depending on the domain. A recent systematic review concluded that respondents tend to report more health problems using a one-week compared to a one-day recall period [40].

In Hungary, population reference data have not been available regarding several health domains, such as vision, hearing, breathing, eating, speech, excretion, and sexual activities before the present study. The sensory functions are especially worth highlighting since they cannot be assessed with any other generic PAM currently available in Hungarian. Learning about the prevalence of problems in these health domains in the general population is a great strength of this study. According to the EHIS and other Eurostat data in 2019, 20.1% of the Hungarian population had problems with walking, 16.6% with seeing, 17.9% with hearing, and 24.9% with usual activities [36, 41, 42]. In this study’s sample, corresponding proportions reached 21.8%, 27.8%, 16.0%, and 21.7%, respectively, meaning that mobility and hearing closely approximate the population values, while respondents had somewhat more problems with vision and fewer problems with usual activities. It is important to note that the questions about experiencing these health problems were differently worded compared to the 15D. No population-level data can be found regarding breathing, eating, speech, excretion, or sexual activities. In this regard, our study offers new information about the Hungarian population’s health status that can also be used to inform public health programs. These findings on the above-mentioned health domains may provide an essential benchmark in cost-effectiveness analyses in several chronic health conditions, for instance, the breathing domain can be beneficial in asthma or chronic obstructive pulmonary disease (COPD), vision in eye diseases, and hearing in hearing impairments.

One may hypothesize that due to the higher prevalence of certain chronic diseases (e.g., osteoarthritis, cardiovascular diseases, vision and hearing impairment, dementia) and gradual decline in functioning, the general health status of the elderly is lower than that of their younger counterparts. However, mean 15D index values in this study showed a significant increase with age, reaching their maximum in the 45–54 age group, then began to decrease in the older age groups. An increase with age in the frequency of any problems was observed in a total of five domains out of 15. To derive index values, 15D responses are weighted with preferences from the value set, therefore both the proportions of problems reported by a population and the domains’ importance order in a value set influence these findings. Earlier, a similar trend was observed mainly in the mental domains of various health status measures in other studies [31, 43–45]. This also applies to this research since the younger generations had more problems and more severe ones with mental function, depression, and distress.

There are certain limitations to be considered. First, 71.5% of the sample reported having a physician-diagnosed chronic illness, while that proportion only reached 48.0% in the Hungarian general population according to the EHIS in 2019 [36]. This difference is likely attributable to the different number of items in the disease lists, i.e., our health conditions list was considerably more detailed. The EHIS asked respondents about 23 physical and only 7 mental health conditions, while our questionnaire included 32 physical and 24 mental health conditions, considering several addictions to be mental conditions as well (e.g., smoking, prescription drugs) following the DSM-5. Second, applying the Norwegian country-specific 15D value set on a Hungarian general population sample is a limitation as it is based on the preferences of the Norwegian general population. So far, national value sets in Hungary are only available for the EQ-5D-5L, EQ-5D-3L, and EQ-5D-Y [46, 47]. Third, since the study was conducted among members of an online panel, it might be prone to selection bias, particularly among older generations and low socioeconomic groups. These demographics are often underrepresented among members of such panels, mostly due to the lack of internet access and digital literacy, potentially leading to suboptimal representation of these groups [48, 49]. According to Eurostat, on average 88.6% of the 16 + Hungarian population used the internet in 2021, while only 62.4% of the 65–74-year-old age group did so [50]. Fourth, physical and mental health conditions with relatively low prevalence in the sample could not be included in the modelling, potentially distorting the results. Fifth, the physician-diagnosed clinical conditions were solely self-reported in the study and not confirmed by the medical records of respondents. Finally, the data collection took place during the COVID-19 pandemic, which may have affected the participants’ mental health status, especially younger generations [51]. However, there was a relatively low number of new cases and restrictions in place in Hungary during the study period [52, 53].

In conclusion, this is the first study to present age- and gender-specific population reference values for the 15D generic PAM on a Hungarian representative sample. The results support health technology assessments and allow the monitoring of the general population’s health status and the disease burden of different health conditions.

### Supplementary Information

Below is the link to the electronic supplementary material.

Supplementary file1 (DOCX 1238 KB)

## Data Availability

All data of this study are available from the corresponding author upon reasonable request.
