# Supplementary material for: Hungarian population norms for the 15D generic preference-accompanied health status measure
Source: Qual Life Res. 2023 Sep 14;33(1):87–99. doi: 10.1007/s11136-023-03514-x (PMC10784351; doi:10.1007/s11136-023-03514-x)
Supplement: Supplementary file 1 — Supplementary file1 (DOCX 1238 KB) [file 11136_2023_3514_MOESM1_ESM.docx]

**Supplementary Information**

**Hungarian population norms for the 15D generic preference-accompanied health status measure**

Anna Nikl, Mathieu F. Janssen, Valentin Brodszky, Fanni Rencz

**Correspondence**:

Fanni Rencz

Department of Health Policy
Corvinus University of Budapest
Address: 8 Fővám tér, H-1093 Budapest, Hungary
E-mail: fanni.rencz@uni-corvinus.hu

Online Resource 1 15D population norms by age group (total)

**Online Resource 2** 15D population norms by age group (males)

**Online Resource 3** 15D population norms by age group (females)

**Online Resource 4** Mean level scores in 15D domains

**Online Resource 5** Mean level scores in each domain by age groups

**Online Resource 6** Mean level scores in each domain by gender and age groups

Online resource 1 15D population norms by age group (total)

|  | **18-24** | | **25-34** | | **35-44** | | **45-54** | | **55-64** | | **65+** | | **Total** | |
| --- | --- | --- | --- | --- | --- | --- | --- | --- | --- | --- | --- | --- | --- | --- |
|  | **n** | **%** | **n** | **%** | **n** | **%** | **n** | **%** | **n** | **%** | **n** | **%** | **n** | **%** |
| **Total** | **202** | **10.1** | **441** | **22.1** | **337** | **16.9** | **285** | **14.3** | **337** | **16.9** | **398** | **19.9** | **2000** | **100.0** |
| **Mobility** |  |  |  |  |  |  |  |  |  |  |  |  |  |  |
| I am able to walk normally (without difficulty) indoors, outdoors and on stairs. | 161 | 79.7 | 374 | 84.8 | 291 | 86.4 | 230 | 80.7 | 248 | 73.6 | 261 | 65.6 | 1565 | 78.3 |
| I am able to walk without difficulty indoors, but outdoors and/or on stairs I have slight difficulties. | 22 | 10.9 | 43 | 9.8 | 31 | 9.2 | 37 | 13.0 | 63 | 18.7 | 96 | 24.1 | 292 | 14.6 |
| I am able to walk without help indoors (with or without an appliance), but outdoors and/or on stairs only with considerable difficulty or with help from others. | 10 | 5.0 | 16 | 3.6 | 11 | 3.3 | 15 | 5.3 | 23 | 6.8 | 37 | 9.3 | 112 | 5.6 |
| I am able to walk indoors only with help from others. | 3 | 1.5 | 3 | 0.7 | 4 | 1.2 | 1 | 0.4 | 3 | 0.9 | 2 | 0.5 | 16 | 0.8 |
| I am completely bed-ridden and unable to move about. | 6 | 3.0 | 5 | 1.1 | 0 | 0.0 | 2 | 0.7 | 0 | 0.0 | 2 | 0.5 | 15 | 0.8 |
| **Vision** |  |  |  |  |  |  |  |  |  |  |  |  |  |  |
| I see normally, i.e. I can read newspapers and TV text without difficulty (with or without glasses). | 143 | 70.8 | 357 | 81.0 | 270 | 80.1 | 180 | 63.2 | 218 | 64.7 | 276 | 69.3 | 1444 | 72.2 |
| I can read papers and/or TV text with slight difficulty (with or without glasses). | 33 | 16.3 | 45 | 10.2 | 39 | 11.6 | 80 | 28.1 | 80 | 23.7 | 99 | 24.9 | 376 | 18.8 |
| I can read papers and/or TV text with considerable difficulty (with or without glasses). | 11 | 5.4 | 29 | 6.6 | 19 | 5.6 | 21 | 7.4 | 32 | 9.5 | 19 | 4.8 | 131 | 6.6 |
| I cannot read papers or TV text either with glasses or without, but I can see enough to walk about without guidance. | 6 | 3.0 | 6 | 1.4 | 7 | 2.1 | 3 | 1.1 | 6 | 1.8 | 4 | 1.0 | 32 | 1.6 |
| I cannot see enough to walk about without a guide, i.e. I am almost or completely blind. | 9 | 4.5 | 4 | 0.9 | 2 | 0.6 | 1 | 0.4 | 1 | 0.3 | 0 | 0.0 | 17 | 0.9 |
| **Hearing** |  |  |  |  |  |  |  |  |  |  |  |  |  |  |
| I can hear normally, i.e. normal speech (with or without a hearing aid). | 164 | 81.2 | 392 | 88.9 | 292 | 86.6 | 245 | 86.0 | 281 | 83.4 | 307 | 77.1 | 1681 | 84.1 |
| I hear normal speech with a little difficulty. | 17 | 8.4 | 25 | 5.7 | 27 | 8.0 | 29 | 10.2 | 39 | 11.6 | 70 | 17.6 | 207 | 10.4 |
| I hear normal speech with considerable difficulty; in conversation I need voices to be louder than normal. | 12 | 5.9 | 20 | 4.5 | 16 | 4.7 | 8 | 2.8 | 16 | 4.7 | 17 | 4.3 | 89 | 4.5 |
| I hear even loud voices poorly; I am almost deaf. | 6 | 3.0 | 3 | 0.7 | 1 | 0.3 | 2 | 0.7 | 1 | 0.3 | 4 | 1.0 | 17 | 0.9 |
| I am completely deaf. | 3 | 1.5 | 1 | 0.2 | 1 | 0.3 | 1 | 0.4 | 0 | 0.0 | 0 | 0.0 | 6 | 0.3 |
| **Breathing** |  |  |  |  |  |  |  |  |  |  |  |  |  |  |
| I am able to breathe normally, i.e. with no shortness of breath or other breathing difficulty. | 156 | 77.2 | 347 | 78.7 | 261 | 77.4 | 214 | 75.1 | 227 | 67.4 | 234 | 58.8 | 1439 | 72.0 |
| I have shortness of breath during heavy work or sports, or when walking briskly on flat ground or slightly uphill. | 27 | 13.4 | 66 | 15.0 | 55 | 16.3 | 55 | 19.3 | 81 | 24.0 | 128 | 32.2 | 412 | 20.6 |
| I have shortness of breath when walking on flat ground at the same speed as others my age. | 16 | 7.9 | 21 | 4.8 | 11 | 3.3 | 11 | 3.9 | 15 | 4.5 | 24 | 6.0 | 98 | 4.9 |
| I get shortness of breath even after light activity, e.g. washing or dressing myself. | 1 | 0.5 | 1 | 0.2 | 6 | 1.8 | 2 | 0.7 | 10 | 3.0 | 9 | 2.3 | 29 | 1.5 |
| I have breathing difficulties almost all the time, even when resting. | 2 | 1.0 | 6 | 1.4 | 4 | 1.2 | 3 | 1.1 | 4 | 1.2 | 3 | 0.8 | 22 | 1.1 |
| **Sleeping** |  |  |  |  |  |  |  |  |  |  |  |  |  |  |
| I am able to sleep normally, i.e. I have no problems with sleeping | 102 | 50.5 | 223 | 50.6 | 173 | 51.3 | 148 | 51.9 | 150 | 44.5 | 189 | 47.5 | 985 | 49.3 |
| I have slight problems with sleeping, e.g. difficulty in falling asleep, or sometimes waking at night. | 58 | 28.7 | 148 | 33.6 | 107 | 31.8 | 91 | 31.9 | 125 | 37.1 | 150 | 37.7 | 679 | 34.0 |
| I have moderate problems with sleeping, e.g. disturbed sleep, or feeling I have not slept enough. | 32 | 15.8 | 58 | 13.2 | 45 | 13.4 | 35 | 12.3 | 45 | 13.4 | 42 | 10.6 | 257 | 12.9 |
| I have great problems with sleeping, e.g. having to use sleeping pills often or routinely, or usually waking at night and/or too early in the morning. | 5 | 2.5 | 10 | 2.3 | 12 | 3.6 | 10 | 3.5 | 12 | 3.6 | 16 | 4.0 | 65 | 3.3 |
| I suffer severe sleeplessness, e.g. sleep is almost impossible even with full use of sleeping pills, or staying awake most of the night. | 5 | 2.5 | 2 | 0.5 | 0 | 0.0 | 1 | 0.4 | 5 | 1.5 | 1 | 0.3 | 14 | 0.7 |
| **Eating** |  |  |  |  |  |  |  |  |  |  |  |  |  |  |
| I am able to eat normally, i.e. with no help from others | 176 | 87.1 | 411 | 93.2 | 315 | 93.5 | 274 | 96.1 | 324 | 96.1 | 390 | 98.0 | 1890 | 94.5 |
| I am able to eat by myself with minor difficulty (e.g. slowly, clumsily, shakily, or with special appliances). | 10 | 5.0 | 13 | 2.9 | 11 | 3.3 | 7 | 2.5 | 9 | 2.7 | 7 | 1.8 | 57 | 2.9 |
| I need some help from another person in eating. | 9 | 4.5 | 12 | 2.7 | 9 | 2.7 | 3 | 1.1 | 3 | 0.9 | 1 | 0.3 | 37 | 1.9 |
| I am unable to eat by myself at all, so I must be fed by another person. | 5 | 2.5 | 4 | 0.9 | 2 | 0.6 | 1 | 0.4 | 1 | 0.3 | 0 | 0.0 | 13 | 0.7 |
| I am unable to eat at all, so I am fed either by tube or intravenously. | 2 | 1.0 | 1 | 0.2 | 0 | 0.0 | 0 | 0.0 | 0 | 0.0 | 0 | 0.0 | 3 | 0.2 |
| **Speech** |  |  |  |  |  |  |  |  |  |  |  |  |  |  |
| I am able to speak normally, i.e. clearly, audibly and fluently. | 156 | 77.2 | 387 | 87.8 | 307 | 91.1 | 266 | 93.3 | 316 | 93.8 | 378 | 95.0 | 1810 | 90.5 |
| I have slight speech difficulties, e.g. occasional fumbling for words, mumbling, or changes of pitch. | 31 | 15.3 | 28 | 6.3 | 17 | 5.0 | 17 | 6.0 | 17 | 5.0 | 17 | 4.3 | 127 | 6.4 |
| I can make myself understood, but my speech is e.g. disjointed, faltering, stuttering or stammering. | 10 | 5.0 | 18 | 4.1 | 11 | 3.3 | 2 | 0.7 | 2 | 0.6 | 1 | 0.3 | 44 | 2.2 |
| Most people have great difficulty understanding my speech. | 3 | 1.5 | 4 | 0.9 | 2 | 0.6 | 0 | 0.0 | 2 | 0.6 | 2 | 0.5 | 13 | 0.7 |
| I can only make myself understood by gestures. | 2 | 1.0 | 4 | 0.9 | 0 | 0.0 | 0 | 0.0 | 0 | 0.0 | 0 | 0.0 | 6 | 0.3 |
| **Excretion** |  |  |  |  |  |  |  |  |  |  |  |  |  |  |
| My bladder and bowel work normally and without problems. | 154 | 76.2 | 355 | 80.5 | 261 | 77.4 | 233 | 81.8 | 236 | 70.0 | 254 | 63.8 | 1493 | 74.7 |
| I have slight problems with my bladder and/or bowel function, e.g. difficulties with urination, or loose or hard bowels. | 28 | 13.9 | 61 | 13.8 | 62 | 18.4 | 41 | 14.4 | 83 | 24.6 | 125 | 31.4 | 400 | 20.0 |
| I have marked problems with my bladder and/or bowel function, e.g. occasional 'accidents', or severe constipation or diarrhea. | 10 | 5.0 | 15 | 3.4 | 12 | 3.6 | 9 | 3.2 | 13 | 3.9 | 17 | 4.3 | 76 | 3.8 |
| I have serious problems with my bladder and/or bowel function, e.g. routine 'accidents', or need of catheterization or enemas. | 4 | 2.0 | 7 | 1.6 | 2 | 0.6 | 2 | 0.7 | 2 | 0.6 | 0 | 0.0 | 17 | 0.9 |
| I have no control over my bladder and/or bowel function. | 6 | 3.0 | 3 | 0.7 | 0 | 0.0 | 0 | 0.0 | 3 | 0.9 | 2 | 0.5 | 14 | 0.7 |
| **Usual activities** |  |  |  |  |  |  |  |  |  |  |  |  |  |  |
| I am able to perform my usual activities (e.g. employment, studying, housework, freetime activities) without difficulty | 167 | 82.7 | 372 | 84.4 | 284 | 84.3 | 217 | 76.1 | 250 | 74.2 | 276 | 69.3 | 1566 | 78.3 |
| I am able to perform my usual activities slightly less effectively or with minor difficulty. | 12 | 5.9 | 44 | 10.0 | 34 | 10.1 | 54 | 18.9 | 60 | 17.8 | 94 | 23.6 | 298 | 14.9 |
| I am able to perform my usual activities much less effectively, with considerable difficulty, or not completely | 12 | 5.9 | 17 | 3.9 | 12 | 3.6 | 9 | 3.2 | 20 | 5.9 | 21 | 5.3 | 91 | 4.6 |
| I can only manage a small proportion of my previously usual activities. | 6 | 3.0 | 6 | 1.4 | 6 | 1.8 | 5 | 1.8 | 7 | 2.1 | 7 | 1.8 | 37 | 1.9 |
| I am unable to manage any of my previously usual activities. | 5 | 2.5 | 2 | 0.5 | 1 | 0.3 | 0 | 0.0 | 0 | 0.0 | 0 | 0.0 | 8 | 0.4 |
| **Mental function** |  |  |  |  |  |  |  |  |  |  |  |  |  |  |
| I am able to think clearly and logically, and my memory functions well | 159 | 78.7 | 373 | 84.6 | 292 | 86.6 | 248 | 87.0 | 294 | 87.2 | 329 | 82.7 | 1695 | 84.8 |
| I have slight difficulties in thinking clearly and logically, or my memory sometimes fails me. | 24 | 11.9 | 43 | 9.8 | 30 | 8.9 | 33 | 11.6 | 38 | 11.3 | 63 | 15.8 | 231 | 11.6 |
| I have marked difficulties in thinking clearly and logically, or my memory is somewhat impaired. | 10 | 5.0 | 18 | 4.1 | 11 | 3.3 | 4 | 1.4 | 2 | 0.6 | 4 | 1.0 | 49 | 2.5 |
| I have great difficulties in thinking clearly and logically, or my memory is seriously impaired. | 5 | 2.5 | 5 | 1.1 | 4 | 1.2 | 0 | 0.0 | 3 | 0.9 | 1 | 0.3 | 18 | 0.9 |
| I am permanently confused and disoriented in place and time. | 4 | 2.0 | 2 | 0.5 | 0 | 0.0 | 0 | 0.0 | 0 | 0.0 | 1 | 0.3 | 7 | 0.4 |
| **Discomfort and symptoms** |  |  |  |  |  |  |  |  |  |  |  |  |  |  |
| I have no physical discomfort or symptoms, e.g. pain, ache, nausea, itching etc. | 144 | 71.3 | 309 | 70.1 | 238 | 70.6 | 204 | 71.6 | 224 | 66.5 | 256 | 64.3 | 1375 | 68.8 |
| I have mild physical discomfort or symptoms, e.g. pain, ache, nausea, itching etc. | 34 | 16.8 | 89 | 20.2 | 71 | 21.1 | 60 | 21.1 | 78 | 23.1 | 108 | 27.1 | 440 | 22.0 |
| I have marked physical discomfort or symptoms, e.g. pain, ache, nausea, itching etc. | 14 | 6.9 | 29 | 6.6 | 20 | 5.9 | 16 | 5.6 | 24 | 7.1 | 29 | 7.3 | 132 | 6.6 |
| I have severe physical discomfort or symptoms, e.g. pain, ache, nausea, itching etc. | 5 | 2.5 | 11 | 2.5 | 8 | 2.4 | 5 | 1.8 | 10 | 3.0 | 5 | 1.3 | 44 | 2.2 |
| I have unbearable physical discomfort or symptoms, e.g. pain, ache, nausea, itching etc. | 5 | 2.5 | 3 | 0.7 | 0 | 0.0 | 0 | 0.0 | 1 | 0.3 | 0 | 0.0 | 9 | 0.5 |
| **Depression** |  |  |  |  |  |  |  |  |  |  |  |  |  |  |
| I do not feel at all sad, melancholic or depressed. | 127 | 62.9 | 291 | 66.0 | 209 | 62.0 | 200 | 70.2 | 240 | 71.2 | 310 | 77.9 | 1377 | 68.9 |
| I feel slightly sad, melancholic or depressed. | 43 | 21.3 | 88 | 20.0 | 76 | 22.6 | 62 | 21.8 | 67 | 19.9 | 58 | 14.6 | 394 | 19.7 |
| I feel moderately sad, melancholic or depressed. | 17 | 8.4 | 42 | 9.5 | 35 | 10.4 | 16 | 5.6 | 23 | 6.8 | 23 | 5.8 | 156 | 7.8 |
| I feel very sad, melancholic or depressed. | 9 | 4.5 | 17 | 3.9 | 11 | 3.3 | 5 | 1.8 | 3 | 0.9 | 6 | 1.5 | 51 | 2.6 |
| I feel extremely sad, melancholic or depressed. | 6 | 3.0 | 3 | 0.7 | 6 | 1.8 | 2 | 0.7 | 4 | 1.2 | 1 | 0.3 | 22 | 1.1 |
| **Distress** |  |  |  |  |  |  |  |  |  |  |  |  |  |  |
| I do not feel at all anxious, stressed or nervous. | 106 | 52.5 | 237 | 53.7 | 177 | 52.5 | 163 | 57.2 | 189 | 56.1 | 256 | 64.3 | 1128 | 56.4 |
| I feel slightly anxious, stressed or nervous. | 52 | 25.7 | 130 | 29.5 | 96 | 28.5 | 87 | 30.5 | 114 | 33.8 | 105 | 26.4 | 584 | 29.2 |
| I feel moderately anxious, stressed or nervous. | 28 | 13.9 | 41 | 9.3 | 33 | 9.8 | 22 | 7.7 | 18 | 5.3 | 29 | 7.3 | 171 | 8.6 |
| I feel very anxious, stressed or nervous. | 10 | 5.0 | 23 | 5.2 | 23 | 6.8 | 10 | 3.5 | 12 | 3.6 | 6 | 1.5 | 84 | 4.2 |
| I feel extremely anxious, stressed or nervous. | 6 | 3.0 | 10 | 2.3 | 8 | 2.4 | 3 | 1.1 | 4 | 1.2 | 2 | 0.5 | 33 | 1.7 |
| **Vitality** |  |  |  |  |  |  |  |  |  |  |  |  |  |  |
| I feel healthy and energetic. | 120 | 59.4 | 242 | 54.9 | 170 | 50.4 | 136 | 47.7 | 168 | 49.9 | 179 | 45.0 | 1015 | 50.8 |
| I feel slightly weary, tired or feeble. | 44 | 21.8 | 117 | 26.5 | 115 | 34.1 | 109 | 38.2 | 117 | 34.7 | 157 | 39.4 | 659 | 33.0 |
| I feel moderately weary, tired or feeble. | 23 | 11.4 | 48 | 10.9 | 29 | 8.6 | 24 | 8.4 | 38 | 11.3 | 49 | 12.3 | 211 | 10.6 |
| I feel very weary, tired or feeble, almost exhausted. | 11 | 5.4 | 26 | 5.9 | 17 | 5.0 | 15 | 5.3 | 13 | 3.9 | 12 | 3.0 | 94 | 4.7 |
| I feel extremely weary, tired or feeble, totally exhausted. | 4 | 2.0 | 8 | 1.8 | 6 | 1.8 | 1 | 0.4 | 1 | 0.3 | 1 | 0.3 | 21 | 1.1 |
| **Sexual activity** |  |  |  |  |  |  |  |  |  |  |  |  |  |  |
| My state of health has no adverse effect on my sexual activity. | 162 | 80.2 | 343 | 77.8 | 241 | 71.5 | 193 | 67.7 | 230 | 68.2 | 238 | 59.8 | 1407 | 70.4 |
| My state of health has a slight effect on my sexual activity. | 22 | 10.9 | 53 | 12.0 | 50 | 14.8 | 54 | 18.9 | 59 | 17.5 | 75 | 18.8 | 313 | 15.7 |
| My state of health has a considerable effect on my sexual activity. | 10 | 5.0 | 36 | 8.2 | 28 | 8.3 | 24 | 8.4 | 25 | 7.4 | 38 | 9.5 | 161 | 8.1 |
| My state of health makes sexual activity almost impossible. | 6 | 3.0 | 8 | 1.8 | 10 | 3.0 | 5 | 1.8 | 7 | 2.1 | 10 | 2.5 | 46 | 2.3 |
| My state of health makes sexual activity impossible. | 2 | 1.0 | 1 | 0.2 | 8 | 2.4 | 9 | 3.2 | 16 | 4.7 | 37 | 9.3 | 73 | 3.7 |
| **Mean index values** |  | |  | |  | |  | |  | |  | |  | |

Totals may not add up to 100 % due to rounding.

Online resource 2 15D population norms by age group (males)

|  | **18-24** | | **25-34** | | **35-44** | | **45-54** | | **55-64** | | **65+** | | **Total** | |
| --- | --- | --- | --- | --- | --- | --- | --- | --- | --- | --- | --- | --- | --- | --- |
|  | **n** | **%** | **n** | **%** | **n** | **%** | **n** | **%** | **n** | **%** | **n** | **%** | **n** | **%** |
| **Total** | **85** | **9.9** | **148** | **17.3** | **162** | **18.9** | **131** | **15.3** | **145** | **17.0** | **184** | **21.5** | **855** | **100.0** |
| **Mobility** |  |  |  |  |  |  |  |  |  |  |  |  |  |  |
| I am able to walk normally (without difficulty) indoors, outdoors and on stairs. | 63 | 74.1 | 121 | 81.8 | 139 | 85.8 | 114 | 87.0 | 115 | 79.3 | 133 | 72.3 | 685 | 80.1 |
| I am able to walk without difficulty indoors, but outdoors and/or on stairs I have slight difficulties. | 12 | 14.1 | 17 | 11.5 | 16 | 9.9 | 10 | 7.6 | 22 | 15.2 | 38 | 20.7 | 115 | 13.5 |
| I am able to walk without help indoors (with or without an appliance), but outdoors and/or on stairs only with considerable difficulty or with help from others. | 3 | 3.5 | 6 | 4.1 | 5 | 3.1 | 6 | 4.6 | 7 | 4.8 | 11 | 6.0 | 38 | 4.4 |
| I am able to walk indoors only with help from others. | 3 | 3.5 | 1 | 0.7 | 2 | 1.2 | 1 | 0.8 | 1 | 0.7 | 0 | 0.0 | 8 | 0.9 |
| I am completely bed-ridden and unable to move about. | 4 | 4.7 | 3 | 2.0 | 0 | 0.0 | 0 | 0.0 | 0 | 0.0 | 2 | 1.1 | 9 | 1.1 |
| **Vision** |  |  |  |  |  |  |  |  |  |  |  |  |  |  |
| I see normally, i.e. I can read newspapers and TV text without difficulty (with or without glasses). | 57 | 67.1 | 119 | 80.4 | 132 | 81.5 | 82 | 62.6 | 96 | 66.2 | 130 | 70.7 | 616 | 72.0 |
| I can read papers and/or TV text with slight difficulty (with or without glasses). | 13 | 15.3 | 13 | 8.8 | 17 | 10.5 | 40 | 30.5 | 33 | 22.8 | 44 | 23.9 | 160 | 18.7 |
| I can read papers and/or TV text with considerable difficulty (with or without glasses). | 5 | 5.9 | 13 | 8.8 | 7 | 4.3 | 6 | 4.6 | 12 | 8.3 | 9 | 4.9 | 52 | 6.1 |
| I cannot read papers or TV text either with glasses or without, but I can see enough to walk about without guidance. | 5 | 5.9 | 3 | 2.0 | 5 | 3.1 | 3 | 2.3 | 3 | 2.1 | 1 | 0.5 | 20 | 2.3 |
| I cannot see enough to walk about without a guide, i.e. I am almost or completely blind. | 5 | 5.9 | 0 | 0.0 | 1 | 0.6 | 0 | 0.0 | 1 | 0.7 | 0 | 0.0 | 7 | 0.8 |
| **Hearing** |  |  |  |  |  |  |  |  |  |  |  |  |  |  |
| I can hear normally, i.e. normal speech (with or without a hearing aid). | 63 | 74.1 | 128 | 86.5 | 137 | 84.6 | 115 | 87.8 | 115 | 79.3 | 134 | 72.8 | 692 | 80.9 |
| I hear normal speech with a little difficulty. | 11 | 12.9 | 10 | 6.8 | 15 | 9.3 | 13 | 9.9 | 21 | 14.5 | 42 | 22.8 | 112 | 13.1 |
| I hear normal speech with considerable difficulty; in conversation I need voices to be louder than normal. | 5 | 5.9 | 8 | 5.4 | 10 | 6.2 | 3 | 2.3 | 8 | 5.5 | 8 | 4.3 | 42 | 4.9 |
| I hear even loud voices poorly; I am almost deaf. | 4 | 4.7 | 2 | 1.4 | 0 | 0.0 | 0 | 0.0 | 1 | 0.7 | 0 | 0.0 | 7 | 0.8 |
| I am completely deaf. | 2 | 2.4 | 0 | 0.0 | 0 | 0.0 | 0 | 0.0 | 0 | 0.0 | 0 | 0.0 | 2 | 0.2 |
| **Breathing** |  |  |  |  |  |  |  |  |  |  |  |  |  |  |
| I am able to breathe normally, i.e. with no shortness of breath or other breathing difficulty. | 60 | 70.6 | 116 | 78.4 | 129 | 79.6 | 105 | 80.2 | 105 | 72.4 | 111 | 60.3 | 626 | 73.2 |
| I have shortness of breath during heavy work or sports, or when walking briskly on flat ground or slightly uphill. | 14 | 16.5 | 23 | 15.5 | 24 | 14.8 | 23 | 17.6 | 33 | 22.8 | 59 | 32.1 | 176 | 20.6 |
| I have shortness of breath when walking on flat ground at the same speed as others my age. | 9 | 10.6 | 8 | 5.4 | 6 | 3.7 | 3 | 2.3 | 3 | 2.1 | 11 | 6.0 | 40 | 4.7 |
| I get shortness of breath even after light activity, e.g. washing or dressing myself. | 1 | 1.2 | 1 | 0.7 | 2 | 1.2 | 0 | 0.0 | 2 | 1.4 | 2 | 1.1 | 8 | 0.9 |
| I have breathing difficulties almost all the time, even when resting. | 1 | 1.2 | 0 | 0.0 | 1 | 0.6 | 0 | 0.0 | 2 | 1.4 | 1 | 0.5 | 5 | 0.6 |
| **Sleeping** |  |  |  |  |  |  |  |  |  |  |  |  |  |  |
| I am able to sleep normally, i.e. I have no problems with sleeping | 49 | 57.6 | 76 | 51.4 | 89 | 54.9 | 80 | 61.1 | 73 | 50.3 | 97 | 52.7 | 464 | 54.3 |
| I have slight problems with sleeping, e.g. difficulty in falling asleep, or sometimes waking at night. | 16 | 18.8 | 47 | 31.8 | 53 | 32.7 | 38 | 29.0 | 52 | 35.9 | 69 | 37.5 | 275 | 32.2 |
| I have moderate problems with sleeping, e.g. disturbed sleep, or feeling I have not slept enough. | 16 | 18.8 | 18 | 12.2 | 16 | 9.9 | 13 | 9.9 | 15 | 10.3 | 12 | 6.5 | 90 | 10.5 |
| I have great problems with sleeping, e.g. having to use sleeping pills often or routinely, or usually waking at night and/or too early in the morning. | 2 | 2.4 | 6 | 4.1 | 4 | 2.5 | 0 | 0.0 | 4 | 2.8 | 6 | 3.3 | 22 | 2.6 |
| I suffer severe sleeplessness, e.g. sleep is almost impossible even with full use of sleeping pills, or staying awake most of the night. | 2 | 2.4 | 1 | 0.7 | 0 | 0.0 | 0 | 0.0 | 1 | 0.7 | 0 | 0.0 | 4 | 0.5 |
| **Eating** |  |  |  |  |  |  |  |  |  |  |  |  |  |  |
| I am able to eat normally, i.e. with no help from others | 68 | 80.0 | 137 | 92.6 | 148 | 91.4 | 126 | 96.2 | 143 | 98.6 | 181 | 98.4 | 803 | 93.9 |
| I am able to eat by myself with minor difficulty (e.g. slowly, clumsily, shakily, or with special appliances). | 7 | 8.2 | 5 | 3.4 | 8 | 4.9 | 3 | 2.3 | 2 | 1.4 | 3 | 1.6 | 28 | 3.3 |
| I need some help from another person in eating. | 5 | 5.9 | 4 | 2.7 | 5 | 3.1 | 2 | 1.5 | 0 | 0.0 | 0 | 0.0 | 16 | 1.9 |
| I am unable to eat by myself at all, so I must be fed by another person. | 4 | 4.7 | 2 | 1.4 | 1 | 0.6 | 0 | 0.0 | 0 | 0.0 | 0 | 0.0 | 7 | 0.8 |
| I am unable to eat at all, so I am fed either by tube or intravenously. | 1 | 1.2 | 0 | 0.0 | 0 | 0.0 | 0 | 0.0 | 0 | 0.0 | 0 | 0.0 | 1 | 0.1 |
| **Speech** |  |  |  |  |  |  |  |  |  |  |  |  |  |  |
| I am able to speak normally, i.e. clearly, audibly and fluently. | 59 | 69.4 | 124 | 83.8 | 143 | 88.3 | 120 | 91.6 | 137 | 94.5 | 176 | 95.7 | 759 | 88.8 |
| I have slight speech difficulties, e.g. occasional fumbling for words, mumbling, or changes of pitch. | 16 | 18.8 | 14 | 9.5 | 11 | 6.8 | 10 | 7.6 | 7 | 4.8 | 7 | 3.8 | 65 | 7.6 |
| I can make myself understood, but my speech is e.g. disjointed, faltering, stuttering or stammering. | 8 | 9.4 | 8 | 5.4 | 6 | 3.7 | 1 | 0.8 | 1 | 0.7 | 1 | 0.5 | 25 | 2.9 |
| Most people have great difficulty understanding my speech. | 1 | 1.2 | 1 | 0.7 | 2 | 1.2 | 0 | 0.0 | 0 | 0.0 | 0 | 0.0 | 4 | 0.5 |
| I can only make myself understood by gestures. | 1 | 1.2 | 1 | 0.7 | 0 | 0.0 | 0 | 0.0 | 0 | 0.0 | 0 | 0.0 | 2 | 0.2 |
| **Excretion** |  |  |  |  |  |  |  |  |  |  |  |  |  |  |
| My bladder and bowel work normally and without problems. | 61 | 71.8 | 121 | 81.8 | 125 | 77.2 | 114 | 87.0 | 104 | 71.7 | 104 | 56.5 | 629 | 73.6 |
| I have slight problems with my bladder and/or bowel function, e.g. difficulties with urination, or loose or hard bowels. | 10 | 11.8 | 17 | 11.5 | 29 | 17.9 | 11 | 8.4 | 38 | 26.2 | 73 | 39.7 | 178 | 20.8 |
| I have marked problems with my bladder and/or bowel function, e.g. occasional 'accidents', or severe constipation or diarrhea. | 7 | 8.2 | 8 | 5.4 | 7 | 4.3 | 5 | 3.8 | 3 | 2.1 | 7 | 3.8 | 37 | 4.3 |
| I have serious problems with my bladder and/or bowel function, e.g. routine 'accidents', or need of catheterization or enemas. | 3 | 3.5 | 2 | 1.4 | 1 | 0.6 | 1 | 0.8 | 0 | 0.0 | 0 | 0.0 | 7 | 0.8 |
| I have no control over my bladder and/or bowel function. | 4 | 4.7 | 0 | 0.0 | 0 | 0.0 | 0 | 0.0 | 0 | 0.0 | 0 | 0.0 | 4 | 0.5 |
| **Usual activities** |  |  |  |  |  |  |  |  |  |  |  |  |  |  |
| I am able to perform my usual activities (e.g. employment, studying, housework, freetime activities) without difficulty | 66 | 77.6 | 123 | 83.1 | 134 | 82.7 | 106 | 80.9 | 117 | 80.7 | 136 | 73.9 | 682 | 79.8 |
| I am able to perform my usual activities slightly less effectively or with minor difficulty. | 6 | 7.1 | 18 | 12.2 | 18 | 11.1 | 21 | 16.0 | 19 | 13.1 | 41 | 22.3 | 123 | 14.4 |
| I am able to perform my usual activities much less effectively, with considerable difficulty, or not completely | 6 | 7.1 | 5 | 3.4 | 6 | 3.7 | 3 | 2.3 | 7 | 4.8 | 5 | 2.7 | 32 | 3.7 |
| I can only manage a small proportion of my previously usual activities. | 4 | 4.7 | 2 | 1.4 | 4 | 2.5 | 1 | 0.8 | 2 | 1.4 | 2 | 1.1 | 15 | 1.8 |
| I am unable to manage any of my previously usual activities. | 3 | 3.5 | 0 | 0.0 | 0 | 0.0 | 0 | 0.0 | 0 | 0.0 | 0 | 0.0 | 3 | 0.4 |
| **Mental function** |  |  |  |  |  |  |  |  |  |  |  |  |  |  |
| I am able to think clearly and logically, and my memory functions well | 60 | 70.6 | 124 | 83.8 | 138 | 85.2 | 113 | 86.3 | 133 | 91.7 | 150 | 81.5 | 718 | 84.0 |
| I have slight difficulties in thinking clearly and logically, or my memory sometimes fails me. | 12 | 14.1 | 13 | 8.8 | 15 | 9.3 | 15 | 11.5 | 12 | 8.3 | 32 | 17.4 | 99 | 11.6 |
| I have marked difficulties in thinking clearly and logically, or my memory is somewhat impaired. | 8 | 9.4 | 7 | 4.7 | 7 | 4.3 | 3 | 2.3 | 0 | 0.0 | 2 | 1.1 | 27 | 3.2 |
| I have great difficulties in thinking clearly and logically, or my memory is seriously impaired. | 4 | 4.7 | 3 | 2.0 | 2 | 1.2 | 0 | 0.0 | 0 | 0.0 | 0 | 0.0 | 9 | 1.1 |
| I am permanently confused and disoriented in place and time. | 1 | 1.2 | 1 | 0.7 | 0 | 0.0 | 0 | 0.0 | 0 | 0.0 | 0 | 0.0 | 2 | 0.2 |
| **Discomfort and symptoms** |  |  |  |  |  |  |  |  |  |  |  |  |  |  |
| I have no physical discomfort or symptoms, e.g. pain, ache, nausea, itching etc. | 63 | 74.1 | 109 | 73.6 | 114 | 70.4 | 99 | 75.6 | 103 | 71.0 | 129 | 70.1 | 617 | 72.2 |
| I have mild physical discomfort or symptoms, e.g. pain, ache, nausea, itching etc. | 8 | 9.4 | 21 | 14.2 | 35 | 21.6 | 28 | 21.4 | 29 | 20.0 | 46 | 25.0 | 167 | 19.5 |
| I have marked physical discomfort or symptoms, e.g. pain, ache, nausea, itching etc. | 7 | 8.2 | 12 | 8.1 | 10 | 6.2 | 3 | 2.3 | 11 | 7.6 | 8 | 4.3 | 51 | 6.0 |
| I have severe physical discomfort or symptoms, e.g. pain, ache, nausea, itching etc. | 4 | 4.7 | 5 | 3.4 | 3 | 1.9 | 1 | 0.8 | 2 | 1.4 | 1 | 0.5 | 16 | 1.9 |
| I have unbearable physical discomfort or symptoms, e.g. pain, ache, nausea, itching etc. | 3 | 3.5 | 1 | 0.7 | 0 | 0.0 | 0 | 0.0 | 0 | 0.0 | 0 | 0.0 | 4 | 0.5 |
| **Depression** |  |  |  |  |  |  |  |  |  |  |  |  |  |  |
| I do not feel at all sad, melancholic or depressed. | 54 | 63.5 | 98 | 66.2 | 107 | 66.0 | 98 | 74.8 | 110 | 75.9 | 155 | 84.2 | 622 | 72.7 |
| I feel slightly sad, melancholic or depressed. | 14 | 16.5 | 23 | 15.5 | 31 | 19.1 | 26 | 19.8 | 27 | 18.6 | 20 | 10.9 | 141 | 16.5 |
| I feel moderately sad, melancholic or depressed. | 6 | 7.1 | 18 | 12.2 | 15 | 9.3 | 5 | 3.8 | 7 | 4.8 | 6 | 3.3 | 57 | 6.7 |
| I feel very sad, melancholic or depressed. | 7 | 8.2 | 9 | 6.1 | 8 | 4.9 | 1 | 0.8 | 1 | 0.7 | 3 | 1.6 | 29 | 3.4 |
| I feel extremely sad, melancholic or depressed. | 4 | 4.7 | 0 | 0.0 | 1 | 0.6 | 1 | 0.8 | 0 | 0.0 | 0 | 0.0 | 6 | 0.7 |
| **Distress** |  |  |  |  |  |  |  |  |  |  |  |  |  |  |
| I do not feel at all anxious, stressed or nervous. | 50 | 58.8 | 91 | 61.5 | 92 | 56.8 | 88 | 67.2 | 100 | 69.0 | 142 | 77.2 | 563 | 65.8 |
| I feel slightly anxious, stressed or nervous. | 17 | 20.0 | 30 | 20.3 | 44 | 27.2 | 36 | 27.5 | 40 | 27.6 | 35 | 19.0 | 202 | 23.6 |
| I feel moderately anxious, stressed or nervous. | 12 | 14.1 | 12 | 8.1 | 12 | 7.4 | 6 | 4.6 | 4 | 2.8 | 6 | 3.3 | 52 | 6.1 |
| I feel very anxious, stressed or nervous. | 3 | 3.5 | 11 | 7.4 | 11 | 6.8 | 0 | 0.0 | 1 | 0.7 | 1 | 0.5 | 27 | 3.2 |
| I feel extremely anxious, stressed or nervous. | 3 | 3.5 | 4 | 2.7 | 3 | 1.9 | 1 | 0.8 | 0 | 0.0 | 0 | 0.0 | 11 | 1.3 |
| **Vitality** |  |  |  |  |  |  |  |  |  |  |  |  |  |  |
| I feel healthy and energetic. | 54 | 63.5 | 96 | 64.9 | 88 | 54.3 | 71 | 54.2 | 77 | 53.1 | 93 | 50.5 | 479 | 56.0 |
| I feel slightly weary, tired or feeble. | 13 | 15.3 | 24 | 16.2 | 52 | 32.1 | 50 | 38.2 | 52 | 35.9 | 71 | 38.6 | 262 | 30.6 |
| I feel moderately weary, tired or feeble. | 11 | 12.9 | 13 | 8.8 | 13 | 8.0 | 3 | 2.3 | 12 | 8.3 | 17 | 9.2 | 69 | 8.1 |
| I feel very weary, tired or feeble, almost exhausted. | 5 | 5.9 | 11 | 7.4 | 6 | 3.7 | 6 | 4.6 | 3 | 2.1 | 3 | 1.6 | 34 | 4.0 |
| I feel extremely weary, tired or feeble, totally exhausted. | 2 | 2.4 | 4 | 2.7 | 3 | 1.9 | 1 | 0.8 | 1 | 0.7 | 0 | 0.0 | 11 | 1.3 |
| **Sexual activity** |  |  |  |  |  |  |  |  |  |  |  |  |  |  |
| My state of health has no adverse effect on my sexual activity. | 63 | 74.1 | 121 | 81.8 | 120 | 74.1 | 91 | 69.5 | 99 | 68.3 | 82 | 44.6 | 576 | 67.4 |
| My state of health has a slight effect on my sexual activity. | 8 | 9.4 | 13 | 8.8 | 19 | 11.7 | 24 | 18.3 | 26 | 17.9 | 49 | 26.6 | 139 | 16.3 |
| My state of health has a considerable effect on my sexual activity. | 9 | 10.6 | 11 | 7.4 | 12 | 7.4 | 10 | 7.6 | 11 | 7.6 | 22 | 12.0 | 75 | 8.8 |
| My state of health makes sexual activity almost impossible. | 4 | 4.7 | 2 | 1.4 | 8 | 4.9 | 1 | 0.8 | 5 | 3.4 | 6 | 3.3 | 26 | 3.0 |
| My state of health makes sexual activity impossible. | 1 | 1.2 | 1 | 0.7 | 3 | 1.9 | 5 | 3.8 | 4 | 2.8 | 25 | 13.6 | 39 | 4.6 |

Totals may not add up to 100 % due to rounding.

Online resource 3 15D population norms by age group (females)

|  | **18-24** | | **25-34** | | **35-44** | | **45-54** | | **55-64** | | **65+** | | **Total** | |
| --- | --- | --- | --- | --- | --- | --- | --- | --- | --- | --- | --- | --- | --- | --- |
|  | **n** | **%** | **n** | **%** | **n** | **%** | **n** | **%** | **n** | **%** | **n** | **%** | **n** | **%** |
| **Total** | **117** | **10.2** | **293** | **25.6** | **175** | **15.3** | **154** | **13.4** | **192** | **16.8** | **214** | **18.7** | **1145** | **100.0** |
| **Mobility** |  |  |  |  |  |  |  |  |  |  |  |  |  |  |
| I am able to walk normally (without difficulty) indoors, outdoors and on stairs. | 98 | 83.8 | 253 | 86.3 | 152 | 86.9 | 116 | 75.3 | 133 | 69.3 | 128 | 59.8 | 880 | 76.9 |
| I am able to walk without difficulty indoors, but outdoors and/or on stairs I have slight difficulties. | 10 | 8.5 | 26 | 8.9 | 15 | 8.6 | 27 | 17.5 | 41 | 21.4 | 58 | 27.1 | 177 | 15.5 |
| I am able to walk without help indoors (with or without an appliance), but outdoors and/or on stairs only with considerable difficulty or with help from others. | 7 | 6.0 | 10 | 3.4 | 6 | 3.4 | 9 | 5.8 | 16 | 8.3 | 26 | 12.1 | 74 | 6.5 |
| I am able to walk indoors only with help from others. | 0 | 0.0 | 2 | 0.7 | 2 | 1.1 | 0 | 0.0 | 2 | 1.0 | 2 | 0.9 | 8 | 0.7 |
| I am completely bed-ridden and unable to move about. | 2 | 1.7 | 2 | 0.7 | 0 | 0.0 | 2 | 1.3 | 0 | 0.0 | 0 | 0.0 | 6 | 0.5 |
| **Vision** |  |  |  |  |  |  |  |  |  |  |  |  |  |  |
| I see normally, i.e. I can read newspapers and TV text without difficulty (with or without glasses). | 86 | 73.5 | 238 | 81.2 | 138 | 78.9 | 98 | 63.6 | 122 | 63.5 | 146 | 68.2 | 828 | 72.3 |
| I can read papers and/or TV text with slight difficulty (with or without glasses). | 20 | 17.1 | 32 | 10.9 | 22 | 12.6 | 40 | 26.0 | 47 | 24.5 | 55 | 25.7 | 216 | 18.9 |
| I can read papers and/or TV text with considerable difficulty (with or without glasses). | 6 | 5.1 | 16 | 5.5 | 12 | 6.9 | 15 | 9.7 | 20 | 10.4 | 10 | 4.7 | 79 | 6.9 |
| I cannot read papers or TV text either with glasses or without, but I can see enough to walk about without guidance. | 1 | 0.9 | 3 | 1.0 | 2 | 1.1 | 0 | 0.0 | 3 | 1.6 | 3 | 1.4 | 12 | 1.0 |
| I cannot see enough to walk about without a guide, i.e. I am almost or completely blind. | 4 | 3.4 | 4 | 1.4 | 1 | 0.6 | 1 | 0.6 | 0 | 0.0 | 0 | 0.0 | 10 | 0.9 |
| **Hearing** |  |  |  |  |  |  |  |  |  |  |  |  |  |  |
| I can hear normally, i.e. normal speech (with or without a hearing aid). | 101 | 86.3 | 264 | 90.1 | 155 | 88.6 | 130 | 84.4 | 166 | 86.5 | 173 | 80.8 | 989 | 86.4 |
| I hear normal speech with a little difficulty. | 6 | 5.1 | 15 | 5.1 | 12 | 6.9 | 16 | 10.4 | 18 | 9.4 | 28 | 13.1 | 95 | 8.3 |
| I hear normal speech with considerable difficulty; in conversation I need voices to be louder than normal. | 7 | 6.0 | 12 | 4.1 | 6 | 3.4 | 5 | 3.2 | 8 | 4.2 | 9 | 4.2 | 47 | 4.1 |
| I hear even loud voices poorly; I am almost deaf. | 2 | 1.7 | 1 | 0.3 | 1 | 0.6 | 2 | 1.3 | 0 | 0.0 | 4 | 1.9 | 10 | 0.9 |
| I am completely deaf. | 1 | 0.9 | 1 | 0.3 | 1 | 0.6 | 1 | 0.6 | 0 | 0.0 | 0 | 0.0 | 4 | 0.3 |
| **Breathing** |  |  |  |  |  |  |  |  |  |  |  |  |  |  |
| I am able to breathe normally, i.e. with no shortness of breath or other breathing difficulty. | 96 | 82.1 | 231 | 78.8 | 132 | 75.4 | 109 | 70.8 | 122 | 63.5 | 123 | 57.5 | 813 | 71.0 |
| I have shortness of breath during heavy work or sports, or when walking briskly on flat ground or slightly uphill. | 13 | 11.1 | 43 | 14.7 | 31 | 17.7 | 32 | 20.8 | 48 | 25.0 | 69 | 32.2 | 236 | 20.6 |
| I have shortness of breath when walking on flat ground at the same speed as others my age. | 7 | 6.0 | 13 | 4.4 | 5 | 2.9 | 8 | 5.2 | 12 | 6.3 | 13 | 6.1 | 58 | 5.1 |
| I get shortness of breath even after light activity, e.g. washing or dressing myself. | 0 | 0.0 | 0 | 0.0 | 4 | 2.3 | 2 | 1.3 | 8 | 4.2 | 7 | 3.3 | 21 | 1.8 |
| I have breathing difficulties almost all the time, even when resting. | 1 | 0.9 | 6 | 2.0 | 3 | 1.7 | 3 | 1.9 | 2 | 1.0 | 2 | 0.9 | 17 | 1.5 |
| **Sleeping** |  |  |  |  |  |  |  |  |  |  |  |  |  |  |
| I am able to sleep normally, i.e. I have no problems with sleeping | 53 | 45.3 | 147 | 50.2 | 84 | 48.0 | 68 | 44.2 | 77 | 40.1 | 92 | 43.0 | 521 | 45.5 |
| I have slight problems with sleeping, e.g. difficulty in falling asleep, or sometimes waking at night. | 42 | 35.9 | 101 | 34.5 | 54 | 30.9 | 53 | 34.4 | 73 | 38.0 | 81 | 37.9 | 404 | 35.3 |
| I have moderate problems with sleeping, e.g. disturbed sleep, or feeling I have not slept enough. | 16 | 13.7 | 40 | 13.7 | 29 | 16.6 | 22 | 14.3 | 30 | 15.6 | 30 | 14.0 | 167 | 14.6 |
| I have great problems with sleeping, e.g. having to use sleeping pills often or routinely, or usually waking at night and/or too early in the morning. | 3 | 2.6 | 4 | 1.4 | 8 | 4.6 | 10 | 6.5 | 8 | 4.2 | 10 | 4.7 | 43 | 3.8 |
| I suffer severe sleeplessness, e.g. sleep is almost impossible even with full use of sleeping pills, or staying awake most of the night. | 3 | 2.6 | 1 | 0.3 | 0 | 0.0 | 1 | 0.6 | 4 | 2.1 | 1 | 0.5 | 10 | 0.9 |
| **Eating** |  |  |  |  |  |  |  |  |  |  |  |  |  |  |
| I am able to eat normally, i.e. with no help from others | 108 | 92.3 | 274 | 93.5 | 167 | 95.4 | 148 | 96.1 | 181 | 94.3 | 209 | 97.7 | 1087 | 94.9 |
| I am able to eat by myself with minor difficulty (e.g. slowly, clumsily, shakily, or with special appliances). | 3 | 2.6 | 8 | 2.7 | 3 | 1.7 | 4 | 2.6 | 7 | 3.6 | 4 | 1.9 | 29 | 2.5 |
| I need some help from another person in eating. | 4 | 3.4 | 8 | 2.7 | 4 | 2.3 | 1 | 0.6 | 3 | 1.6 | 1 | 0.5 | 21 | 1.8 |
| I am unable to eat by myself at all, so I must be fed by another person. | 1 | 0.9 | 2 | 0.7 | 1 | 0.6 | 1 | 0.6 | 1 | 0.5 | 0 | 0.0 | 6 | 0.5 |
| I am unable to eat at all, so I am fed either by tube or intravenously. | 1 | 0.9 | 1 | 0.3 | 0 | 0.0 | 0 | 0.0 | 0 | 0.0 | 0 | 0.0 | 2 | 0.2 |
| **Speech** |  |  |  |  |  |  |  |  |  |  |  |  |  |  |
| I am able to speak normally, i.e. clearly, audibly and fluently. | 97 | 82.9 | 263 | 89.8 | 164 | 93.7 | 146 | 94.8 | 179 | 93.2 | 202 | 94.4 | 1051 | 91.8 |
| I have slight speech difficulties, e.g. occasional fumbling for words, mumbling, or changes of pitch. | 15 | 12.8 | 14 | 4.8 | 6 | 3.4 | 7 | 4.5 | 10 | 5.2 | 10 | 4.7 | 62 | 5.4 |
| I can make myself understood, but my speech is e.g. disjointed, faltering, stuttering or stammering. | 2 | 1.7 | 10 | 3.4 | 5 | 2.9 | 1 | 0.6 | 1 | 0.5 | 0 | 0.0 | 19 | 1.7 |
| Most people have great difficulty understanding my speech. | 2 | 1.7 | 3 | 1.0 | 0 | 0.0 | 0 | 0.0 | 2 | 1.0 | 2 | 0.9 | 9 | 0.8 |
| I can only make myself understood by gestures. | 1 | 0.9 | 3 | 1.0 | 0 | 0.0 | 0 | 0.0 | 0 | 0.0 | 0 | 0.0 | 4 | 0.3 |
| **Excretion** |  |  |  |  |  |  |  |  |  |  |  |  |  |  |
| My bladder and bowel work normally and without problems. | 93 | 79.5 | 234 | 79.9 | 136 | 77.7 | 119 | 77.3 | 132 | 68.8 | 150 | 70.1 | 864 | 75.5 |
| I have slight problems with my bladder and/or bowel function, e.g. difficulties with urination, or loose or hard bowels. | 18 | 15.4 | 44 | 15.0 | 33 | 18.9 | 30 | 19.5 | 45 | 23.4 | 52 | 24.3 | 222 | 19.4 |
| I have marked problems with my bladder and/or bowel function, e.g. occasional 'accidents', or severe constipation or diarrhea. | 3 | 2.6 | 7 | 2.4 | 5 | 2.9 | 4 | 2.6 | 10 | 5.2 | 10 | 4.7 | 39 | 3.4 |
| I have serious problems with my bladder and/or bowel function, e.g. routine 'accidents', or need of catheterization or enemas. | 1 | 0.9 | 5 | 1.7 | 1 | 0.6 | 1 | 0.6 | 2 | 1.0 | 0 | 0.0 | 10 | 0.9 |
| I have no control over my bladder and/or bowel function. | 2 | 1.7 | 3 | 1.0 | 0 | 0.0 | 0 | 0.0 | 3 | 1.6 | 2 | 0.9 | 10 | 0.9 |
| **Usual activities** |  |  |  |  |  |  |  |  |  |  |  |  |  |  |
| I am able to perform my usual activities (e.g. employment, studying, housework, freetime activities) without difficulty | 101 | 86.3 | 249 | 85.0 | 150 | 85.7 | 111 | 72.1 | 133 | 69.3 | 140 | 65.4 | 884 | 77.2 |
| I am able to perform my usual activities slightly less effectively or with minor difficulty. | 6 | 5.1 | 26 | 8.9 | 16 | 9.1 | 33 | 21.4 | 41 | 21.4 | 53 | 24.8 | 175 | 15.3 |
| I am able to perform my usual activities much less effectively, with considerable difficulty, or not completely | 6 | 5.1 | 12 | 4.1 | 6 | 3.4 | 6 | 3.9 | 13 | 6.8 | 16 | 7.5 | 59 | 5.2 |
| I can only manage a small proportion of my previously usual activities. | 2 | 1.7 | 4 | 1.4 | 2 | 1.1 | 4 | 2.6 | 5 | 2.6 | 5 | 2.3 | 22 | 1.9 |
| I am unable to manage any of my previously usual activities. | 2 | 1.7 | 2 | 0.7 | 1 | 0.6 | 0 | 0.0 | 0 | 0.0 | 0 | 0.0 | 5 | 0.4 |
| **Mental function** |  |  |  |  |  |  |  |  |  |  |  |  |  |  |
| I am able to think clearly and logically, and my memory functions well | 99 | 84.6 | 249 | 85.0 | 154 | 88.0 | 135 | 87.7 | 161 | 83.9 | 179 | 83.6 | 977 | 85.3 |
| I have slight difficulties in thinking clearly and logically, or my memory sometimes fails me. | 12 | 10.3 | 30 | 10.2 | 15 | 8.6 | 18 | 11.7 | 26 | 13.5 | 31 | 14.5 | 132 | 11.5 |
| I have marked difficulties in thinking clearly and logically, or my memory is somewhat impaired. | 2 | 1.7 | 11 | 3.8 | 4 | 2.3 | 1 | 0.6 | 2 | 1.0 | 2 | 0.9 | 22 | 1.9 |
| I have great difficulties in thinking clearly and logically, or my memory is seriously impaired. | 1 | 0.9 | 2 | 0.7 | 2 | 1.1 | 0 | 0.0 | 3 | 1.6 | 1 | 0.5 | 9 | 0.8 |
| I am permanently confused and disoriented in place and time. | 3 | 2.6 | 1 | 0.3 | 0 | 0.0 | 0 | 0.0 | 0 | 0.0 | 1 | 0.5 | 5 | 0.4 |
| **Discomfort and symptoms** |  |  |  |  |  |  |  |  |  |  |  |  |  |  |
| I have no physical discomfort or symptoms, e.g. pain, ache, nausea, itching etc. | 81 | 69.2 | 200 | 68.3 | 124 | 70.9 | 105 | 68.2 | 121 | 63.0 | 127 | 59.3 | 758 | 66.2 |
| I have mild physical discomfort or symptoms, e.g. pain, ache, nausea, itching etc. | 26 | 22.2 | 68 | 23.2 | 36 | 20.6 | 32 | 20.8 | 49 | 25.5 | 62 | 29.0 | 273 | 23.8 |
| I have marked physical discomfort or symptoms, e.g. pain, ache, nausea, itching etc. | 7 | 6.0 | 17 | 5.8 | 10 | 5.7 | 13 | 8.4 | 13 | 6.8 | 21 | 9.8 | 81 | 7.1 |
| I have severe physical discomfort or symptoms, e.g. pain, ache, nausea, itching etc. | 1 | 0.9 | 6 | 2.0 | 5 | 2.9 | 4 | 2.6 | 8 | 4.2 | 4 | 1.9 | 28 | 2.4 |
| I have unbearable physical discomfort or symptoms, e.g. pain, ache, nausea, itching etc. | 2 | 1.7 | 2 | 0.7 | 0 | 0.0 | 0 | 0.0 | 1 | 0.5 | 0 | 0.0 | 5 | 0.4 |
| **Depression** |  |  |  |  |  |  |  |  |  |  |  |  |  |  |
| I do not feel at all sad, melancholic or depressed. | 73 | 62.4 | 193 | 65.9 | 102 | 58.3 | 102 | 66.2 | 130 | 67.7 | 155 | 72.4 | 755 | 65.9 |
| I feel slightly sad, melancholic or depressed. | 29 | 24.8 | 65 | 22.2 | 45 | 25.7 | 36 | 23.4 | 40 | 20.8 | 38 | 17.8 | 253 | 22.1 |
| I feel moderately sad, melancholic or depressed. | 11 | 9.4 | 24 | 8.2 | 20 | 11.4 | 11 | 7.1 | 16 | 8.3 | 17 | 7.9 | 99 | 8.6 |
| I feel very sad, melancholic or depressed. | 2 | 1.7 | 8 | 2.7 | 3 | 1.7 | 4 | 2.6 | 2 | 1.0 | 3 | 1.4 | 22 | 1.9 |
| I feel extremely sad, melancholic or depressed. | 2 | 1.7 | 3 | 1.0 | 5 | 2.9 | 1 | 0.6 | 4 | 2.1 | 1 | 0.5 | 16 | 1.4 |
| **Distress** |  |  |  |  |  |  |  |  |  |  |  |  |  |  |
| I do not feel at all anxious, stressed or nervous. | 56 | 47.9 | 146 | 49.8 | 85 | 48.6 | 75 | 48.7 | 89 | 46.4 | 114 | 53.3 | 565 | 49.3 |
| I feel slightly anxious, stressed or nervous. | 35 | 29.9 | 100 | 34.1 | 52 | 29.7 | 51 | 33.1 | 74 | 38.5 | 70 | 32.7 | 382 | 33.4 |
| I feel moderately anxious, stressed or nervous. | 16 | 13.7 | 29 | 9.9 | 21 | 12.0 | 16 | 10.4 | 14 | 7.3 | 23 | 10.7 | 119 | 10.4 |
| I feel very anxious, stressed or nervous. | 7 | 6.0 | 12 | 4.1 | 12 | 6.9 | 10 | 6.5 | 11 | 5.7 | 5 | 2.3 | 57 | 5.0 |
| I feel extremely anxious, stressed or nervous. | 3 | 2.6 | 6 | 2.0 | 5 | 2.9 | 2 | 1.3 | 4 | 2.1 | 2 | 0.9 | 22 | 1.9 |
| **Vitality** |  |  |  |  |  |  |  |  |  |  |  |  |  |  |
| I feel healthy and energetic. | 66 | 56.4 | 146 | 49.8 | 82 | 46.9 | 65 | 42.2 | 91 | 47.4 | 86 | 40.2 | 536 | 46.8 |
| I feel slightly weary, tired or feeble. | 31 | 26.5 | 93 | 31.7 | 63 | 36.0 | 59 | 38.3 | 65 | 33.9 | 86 | 40.2 | 397 | 34.7 |
| I feel moderately weary, tired or feeble. | 12 | 10.3 | 35 | 11.9 | 16 | 9.1 | 21 | 13.6 | 26 | 13.5 | 32 | 15.0 | 142 | 12.4 |
| I feel very weary, tired or feeble, almost exhausted. | 6 | 5.1 | 15 | 5.1 | 11 | 6.3 | 9 | 5.8 | 10 | 5.2 | 9 | 4.2 | 60 | 5.2 |
| I feel extremely weary, tired or feeble, totally exhausted. | 2 | 1.7 | 4 | 1.4 | 3 | 1.7 | 0 | 0.0 | 0 | 0.0 | 1 | 0.5 | 10 | 0.9 |
| **Sexual activity** |  |  |  |  |  |  |  |  |  |  |  |  |  |  |
| My state of health has no adverse effect on my sexual activity. | 99 | 84.6 | 222 | 75.8 | 121 | 69.1 | 102 | 66.2 | 131 | 68.2 | 156 | 72.9 | 831 | 72.6 |
| My state of health has a slight effect on my sexual activity. | 14 | 12.0 | 40 | 13.7 | 31 | 17.7 | 30 | 19.5 | 33 | 17.2 | 26 | 12.1 | 174 | 15.2 |
| My state of health has a considerable effect on my sexual activity. | 1 | 0.9 | 25 | 8.5 | 16 | 9.1 | 14 | 9.1 | 14 | 7.3 | 16 | 7.5 | 86 | 7.5 |
| My state of health makes sexual activity almost impossible. | 2 | 1.7 | 6 | 2.0 | 2 | 1.1 | 4 | 2.6 | 2 | 1.0 | 4 | 1.9 | 20 | 1.7 |
| My state of health makes sexual activity impossible. | 1 | 0.9 | 0 | 0.0 | 5 | 2.9 | 4 | 2.6 | 12 | 6.3 | 12 | 5.6 | 34 | 3.0 |

Totals may not add up to 100 % due to rounding.

Online resource 4 Mean level scores in 15D domains


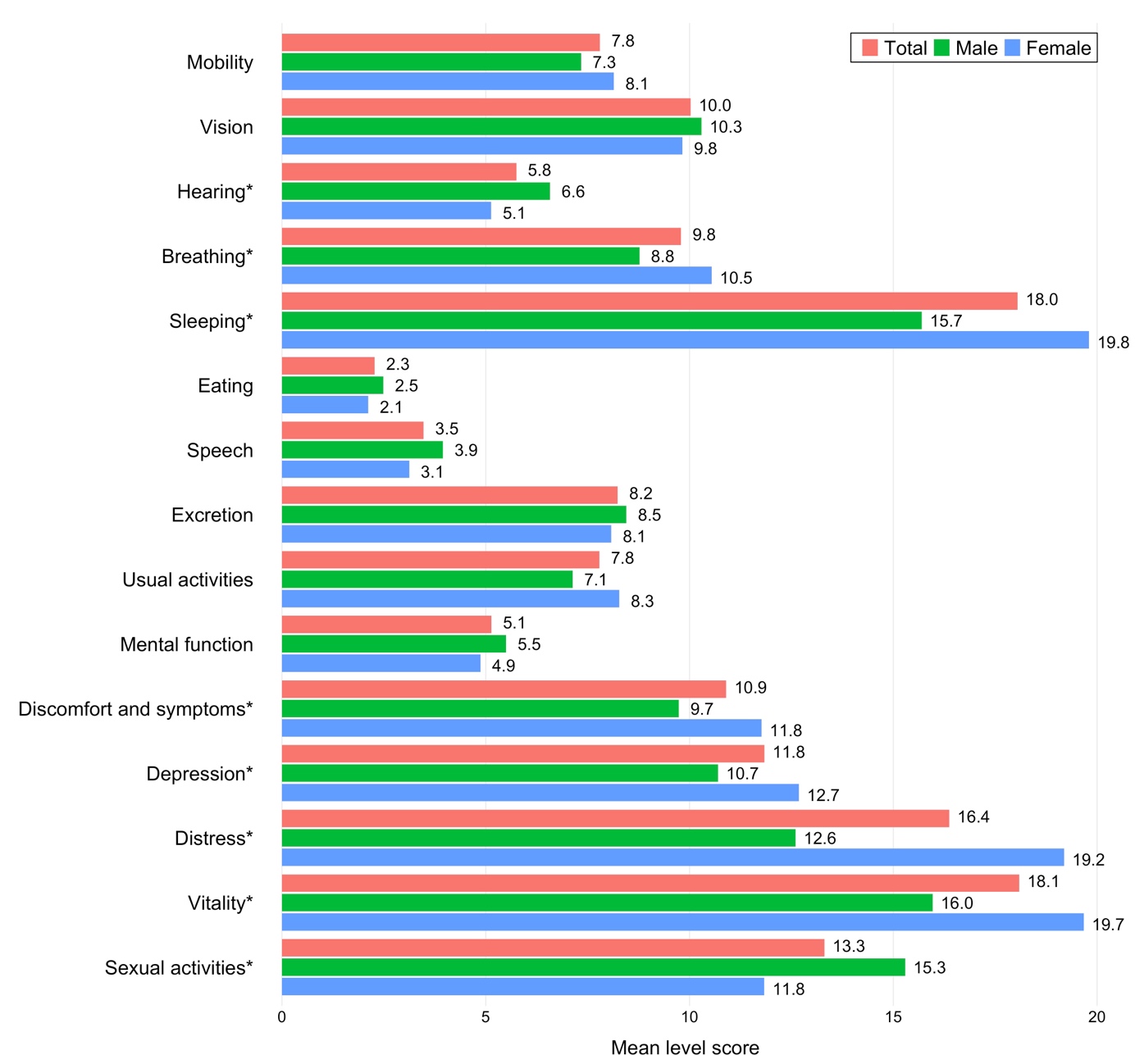


Student’s t-test was performed to assess the mean level score difference between genders. All domains where p-value was <0.05 are marked with asterisks.

The LS ranges from 0 to 100.

Online resource 5 Mean level scores in each domain by age groups


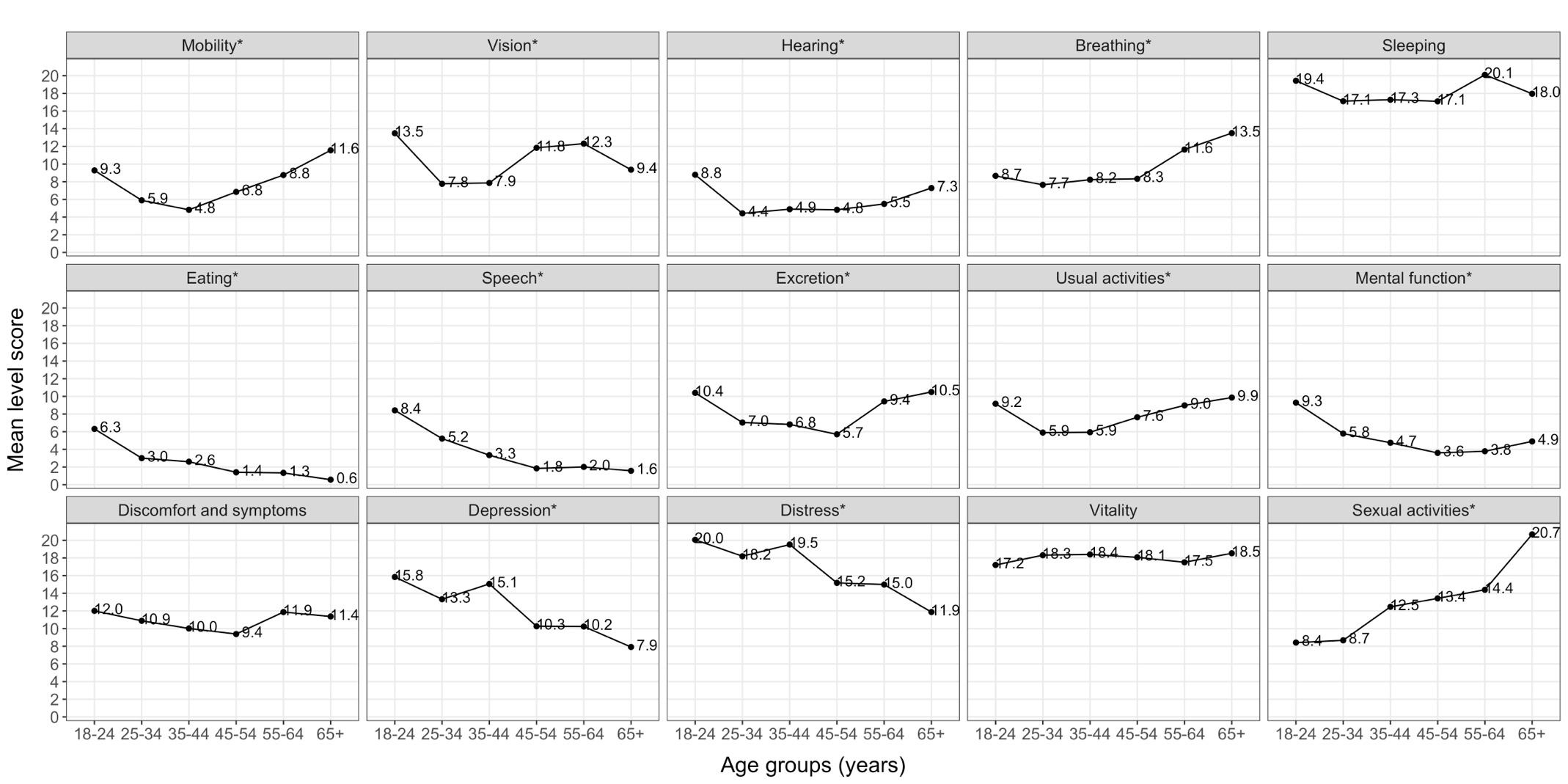


Analysis of variance was performed to assess the mean level score difference between age groups. All domains where p-value was <0.05 are marked with asterisks.

The LS ranges from 0 to 100.

Online resource 6 Mean level scores in each domain by gender and age groups


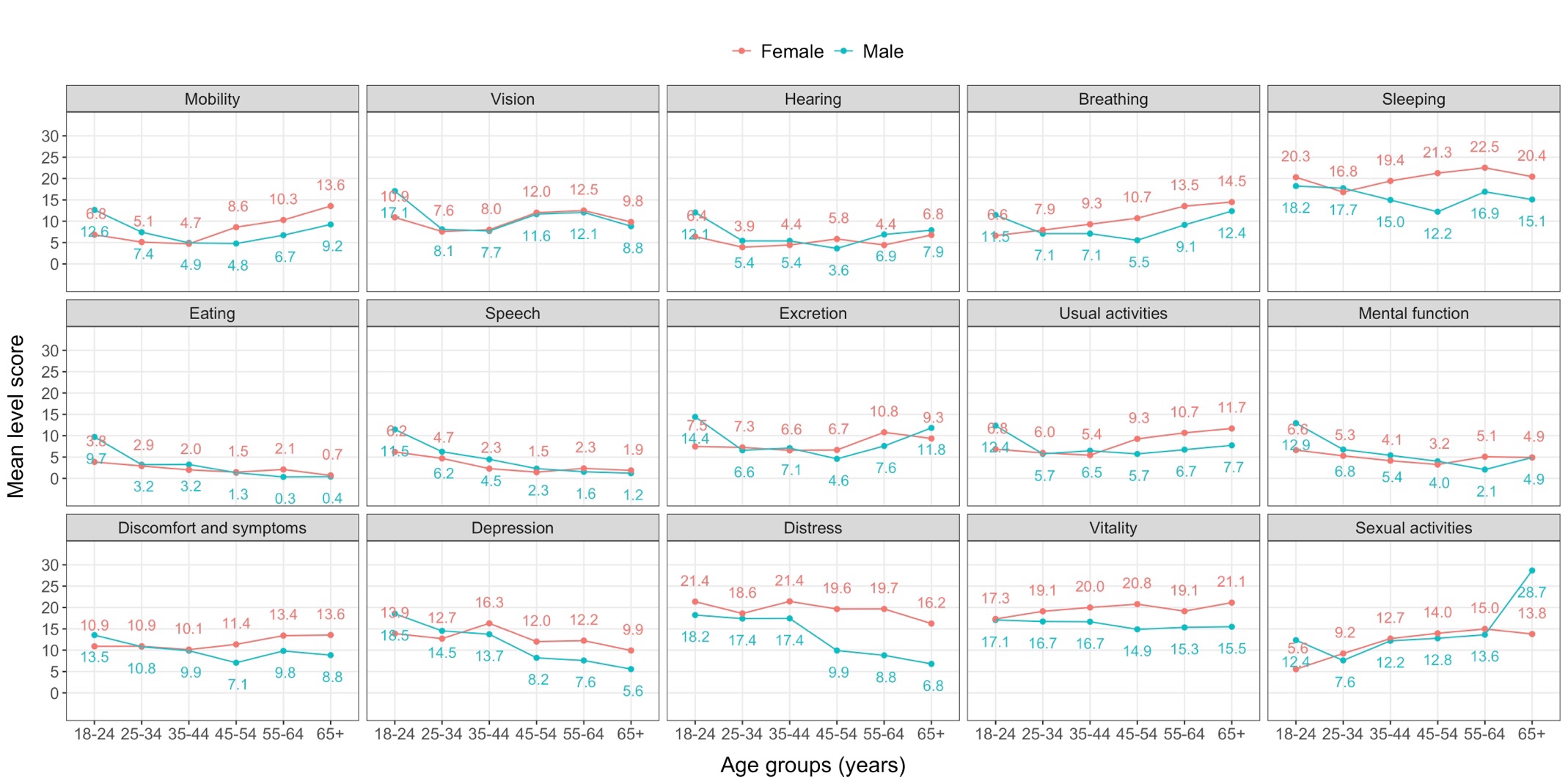


The LS ranges from 0 to 100.
